# Relationship Between Serum Levels of Unsaturated Fatty Acids and Type 2 Diabetes Mellitus: A Cross‐Sectional Analysis of NHANES 2003–2004 and 2011–2012

**DOI:** 10.1155/jdr/1153035

**Published:** 2026-03-01

**Authors:** Shan Liu, Ying Liu, Lipeng Liu, Fengxia Lv, Huijuan Wang, Xiuyun Zhang, Shuxia Yue, Liwen Zhang, Jin Zhou

**Affiliations:** ^1^ Department of Endocrinology, The Second Hospital of Shijiazhuang, Shijiazhuang, Hebei, China; ^2^ Obstetrical Department VIII, The Fourth Hospital of Shijiazhuang, Shijiazhuang, Hebei, China; ^3^ Hebei Key Laboratory of Basic Medicine for Diabetes, The Second Hospital of Shijiazhuang, Shijiazhuang, Hebei, China; ^4^ College of Veterinary, Hebei Agricultural University, Baoding, China, hebau.edu.cn; ^5^ Department of Epidemiology and Statistics, School of Public Health, Hebei Medical University, Hebei Key Laboratory of Environment and Human Health, Shijiazhuang, Hebei, China, hebmu.edu.cn; ^6^ Health Care Center, The First Hospital of Hebei Medical University, Shijiazhuang, Hebei, China, hebmu.edu.cn

**Keywords:** monounsaturated fatty acids, polyunsaturated fatty acids, prevalence, Type 2 diabetes mellitus

## Abstract

**Objective:**

The role of serum fatty acids in Type 2 diabetes mellitus (T2DM) remains unclear. We aimed to assess and compare the associations of multiple serum unsaturated fatty acids with the prevalence of T2DM to elucidate their heterogeneous relationship profiles.

**Methods:**

This study used the data from the National Health and Nutrition Examination Surveys (2003–2004 and 2011–2012). Weighted proportional and multivariate logistic regression analyses were conducted to evaluate the associations of serum polyunsaturated fatty acids (PUFAs) and monounsaturated fatty acids (MUFAs) with T2DM status and to adjust for potential confounders.

**Results:**

The study included 3760 individuals. Results of the multivariate logistic regression analyses showed that among the serum PUFAs, docosapentaenoic acid (DPAn3) (22:5 n3) was associated with lower odds of T2DM (odds ratio [OR]: 0.595, 95% CI: 0.375–0.942, *P*_trend_ = 0.028). Arachidonic acid (AA) (20:4(20: 4 n6) and linoleic acid (LA) (18:2 n6) were negatively associated with T2DM prevalence (OR in quintile 5: AA [20:4 n6] = 0.396, 95% CI: 0.232–0.674, *P*_trend_ = 0.002; LA (18:2 n6) = 0.277, 95% CI: 0.163–0.472, *P*_trend_ < 0.001). Moreover, among the MUFAs, oleic acid (OA) (18:1 n9) and eicosenoic acid (EA) (20:1 n9) were associated with increased odds of T2DM, whereas nervonic acid (NRA) (24:1 n9) was associated with decreased odds of T2DM. Furthermore, nonlinear analyses showed that the n‐6 PUFAs dihomo‐*γ*‐linolenic acid (DGLA) (20:3 n6) and AA (20:4 n6), as well as the n‐9 MUFA EA (20:1 n9), had nonlinear associations with T2DM.

**Conclusions:**

Our findings suggest that several serum n‐3 and n‐6 PUFAs are inversely associated with T2DM prevalence, whereas some n‐9 MUFAs are positively associated with T2DM prevalence. These findings provide comprehensive descriptive data on the serum fatty acid profile in relation to T2DM and underscore the heterogeneity of associations across individual fatty acid species.

## 1. Introduction

Diabetes mellitus is a group of metabolic disorders characterized by hyperglycemia arising from multiple etiologies. The major subtype is Type 2 diabetes mellitus (T2DM), which accounts for more than 90% of the diabetic population [1]. Both microvascular and macrovascular complications are primary contributors to morbidity and mortality in patients with T2DM, imposing a substantial financial burden on many countries [2]. Therefore, implementing effective preventive measures is crucial.

Unsaturated fatty acids (UFAs), including polyunsaturated fatty acids (PUFAs) and monounsaturated fatty acids (MUFAs), have received considerable attention in diabetes research [3, 4]. PUFAs are categorized into omega‐3 (n‐3) and omega‐6 (n‐6 PUFAs) subclasses [5]. Previous studies have shown that certain n‐3 and n‐6 PUFAs play important roles in regulating glucose homeostasis, lipid metabolism, and inflammation [6]. Although some studies have suggested that PUFA intake may help reduce the risk of T2DM [7, 8], others have reported an increased risk [9, 10]. MUFAs, such as the n‐7 and n‐9 subclasses, have also gained increasing research interest. Reports indicate that n‐7 and n‐9 MUFAs participate in the regulation of inflammation and lipid metabolism [11–13], processes that are involved in the onset and progression of diabetes. However, the relationship between UFAs, including both PUFAs and MUFAs, and T2DM remains unclear. Circulating UFAs levels are influenced by multiple factors, including dietary intake, endogenous biosynthesis, and metabolic pathways [14]. For example, PUFAs, such as *α*‐linolenic acid (ALA) (18:3n‐3) and linoleic acid (LA) (18:2n‐6), can be metabolized in vivo to generate further PUFA derivatives [15]. Thus, circulating PUFA levels reflect the combined influence of dietary intake and metabolic conversion [16]. Therefore, objectively measured circulating PUFA concentrations may more accurately represent bioavailable PUFA status than dietary data alone. However, relatively few studies have evaluated the associations between objectively measured circulating PUFAs and MUFAs and T2DM. In our previous study, we explored the relationships between serum PUFA and MUFA levels and prediabetes risk [17]. The role of these major PUFAs in T2DM has yet to be fully clarified, and the contributions of less frequently measured PUFAs and MUFAs also require further investigation.

In this study, we aimed to evaluate the associations of specific serum PUFAs (n‐3 and n‐6) and MUFAs (n‐5, n‐7, and n‐9) with the prevalence of T2DM using data from the National Health and Nutrition Examination Survey (NHANES).

## 2. Methods

### 2.1. Study Population

The NHANES is a systematic national health survey program involving interviews and examinations, including demographic information, dietary data, biological monitoring, and physical assessments. Data were obtained from NHANES cycles 2003–2004 and 2011–2012, which provided serum PUFA measurements (*n* = 19878). After excluding participants younger than 18 years (*n* = 8394), those with missing serum fatty acid values (*n* = 7139), and those diagnosed with prediabetes (*n* = 585), a total of 3760 participants were included in the present analysis.

### 2.2. Assessment of Serum Fatty Acids

Serum fatty acids were quantified using gas chromatography–mass spectrometry (GC‐MS). Esterified fatty acids were hydrolysed primarily from triglycerides, phospholipids, and cholesteryl esters through sequential mineral acid and base treatment in the presence of heat. Total fatty acids were extracted from 100 *μ*L of serum or plasma using hexane, along with an internal standard containing 18 stable isotopically labelled fatty acids to correct for recovery. The extract was derivatized with pentafluorobenzyl bromide in the presence of triethylamine to produce pentafluorobenzyl esters. The reaction mixture was injected into a capillary gas chromatograph column to separate individual fatty acids from other matrix constituents. Detection was performed using electron capture negative‐ion mass spectrometry within 34 min. Quantification was achieved by comparing the analyte peak area in each sample with that of a calibrator solution, with results corrected using the internal standard peak area.

Serum fatty acids included PUFAs (n‐3 and n‐6) and MUFAs (n‐5, n‐7, and n‐9). n‐3 fatty acids included *α*‐linolenic acid (18:3 n‐3) (ALA), eicosapentaenoic acid (20:5 n‐3) (EPA), docosapentaenoic acid (22:5 n‐3) (DPAn3), and docosahexaenoic acid (22:6 n‐3) (DHA). The n‐5 fatty acids included myristoleic acid (14:1 n‐5) (MA). n‐6 fatty acids included linoleic acid (18:2 n‐6) (LA), *γ*‐linolenic acid (18:3 n‐6) (GLA), dihomo‐*γ*‐linolenic acid (20:3 n‐6) (DGLA), arachidonic acid (20:4 n‐6) (AA), eicosadienoic acid (20:2 n‐6) (EDA), docosatetraenoic acid (22:4 n‐6) (DTA), and docosapentaenoic acid (22:5 n‐6) (DPAn6). The n‐7 fatty acids included palmitoleic acid (16:1 n‐7) (PA) and *cis*‐vaccenic acid (18:1 n‐7) (VA). The n‐9 fatty acids included oleic acid (18:1 n‐9) (OA), eicosenoic acid (20:1 n‐9) (EA), and nervonic acid (24:1 n‐9) (NRA).

### 2.3. Diagnosis of T2DM

T2DM was defined as self‐reported physician diagnosis of diabetes, current use of insulin or oral hypoglycemic medications, fasting blood glucose ≥ 7.0 mmol/L (126 mg/dL), 2‐h post–oral glucose tolerance test plasma glucose ≥ 11.1 mmol/L (200 mg/dL), or glycated hemoglobin (HbA1c) ≥ 6.5% (48 mmol/mol).

### 2.4. Assessment of Covariates

Covariates included age (continuous), sex (male, female), race (non‐Hispanic White, others), education (less than high school, high school, college graduate, or above), waist circumference (WC) (continuous), body mass index (BMI) (continuous), family income‐to‐poverty ratio (≤ 1.0, 1.0–3.0, > 3.0), smoking (never, former, or current), drinking (nondrinker, low‐to‐moderate drinker, or heavy drinker), physical activity (no, moderate, or vigorous), hypertension (no or yes), cardiovascular disease (CVD) (no or yes), and cancer (no or yes).

BMI was calculated as weight (kg)/height^2^ (m^2^). Former smokers were individuals who had smoked more than 100 cigarettes in their lifetime but quit. Current smokers had smoked more than 100 cigarettes and currently smoked every day or on some days. Low‐to‐moderate drinkers were defined as < 2 drinks/day for men and < 1 drink/day for women, and heavy drinkers as ≥ 2 drinks/day for men and ≥ 1 drink/day for women. Physical activity was divided as follows: no activity, with almost no physical exercise in the past 30 days; moderate activity, defined as ≥ 10 min of moderate‐intensity exercise in the past 30 days causing slight sweating or slight increases in heart or respiratory rate; vigorous activity, defined as ≥ 10 min of vigorous exercise in the past 30 days causing excessive sweating or marked increases in heart or respiratory rate. Hypertension was defined as average systolic blood pressure ≥ 140 mmHg, average diastolic blood pressure ≥ 90 mmHg, use of antihypertensive medication, or self‐reported physician diagnosis. CVD was determined based on self‐reported physician diagnoses of coronary heart disease, angina pectoris, myocardial infarction, or stroke. Cancer status was defined as a self‐reported physician diagnosis.

### 2.5. Statistical Analysis

Given the complex NHANES sampling design, sample weights, clustering, and stratification were applied. Survey design effects were accounted for using clustering and stratification variables linked to testing weights and demographic datasets from two‐year mobile test centres. Continuous variables were presented as means (95% CI), and age‐adjusted general linear models were used for comparisons between the T2DM and control groups. Categorical variables were expressed as percentages (95% CI), and chi‐square tests were used for group comparisons. To evaluate the association between PUFAs and T2DM prevalence, three multivariate logistic regression models were developed. Model 1 adjusted for age. Model 2 adjusted for age, sex, race, education, BMI, WC, poverty‐income ratio (PIR), smoking, drinking, and physical activity. Model 3 further adjusted for hypertension, CVD, and cancer. Restricted cubic splines (RCSs) were applied to assess possible nonlinear associations between serum PUFAs and T2DM. Analyses were conducted using R software Version 4.1.3. A *p* value < 0.05 was considered statistically significant.

## 3. Results

Table 1 describes the baseline characteristics of the 3760 individuals. Compared with the control group, patients with T2DM tended to be older, have higher WC and BMI, lower education level and family income, lower rates of smoking and drinking, lower physical activity levels, and higher prevalence of hypertension, CVD, and cancer. Baseline characteristics according to quintiles of each PUFA are shown in Tables S1–S17. Several serum PUFAs showed similar characteristics. For example, higher levels of DPAn3 (22:5 n3) and DHA (22:6 n3) were associated with older age, lower BMI and WC, and reduced smoking prevalence. Similarly, higher levels of LA (18:2 n6), EDA (20:2 n6), and NRA (24:1 n9) correlated with lower BMI and WC and lower smoking rates. Higher PA (16:1 n7) and VA (18:1 n7) levels were associated with reduced physical activity and a higher likelihood of hypertension or CVD.

**Table 1:** Baseline characteristics of the 3760 people from the NHANES in 2003–2004 and 2011–2012^a^.

	Total *n* = 3760	Control *n* = 2990	T2DM *n* = 770	*p* value^b^
Male, %	47.9 (46–49.8)	47.1 (45.4‐49.0)	52.0 (46.9–57.0)	0.054
Age, years	46.02 (44.94–47.1)	43.51 (42.35–44.67)	59.95 (58.29‐61.60)	< 0.001
BMI, kg/m^2^	28.29 (27.94–28.64)	27.51 (27.20–27.82)	32.68 (32.05–33.30)	< 0.001
Waist circumference, cm	97.49 (96.5–98.48)	95.24 (94.39–96.09)	110.54 (108.90–112.17)	< 0.001
Non‐Hispanic White, %	69.5 (63.9–74.6)	70.9 (65.6–76.0)	61.7 (52.8–69.9)	0.001
Education				< 0.001
Less than high school, %	17.9 (15.1–21.1)	16.4 (13.5–20.0)	25.8 (22.0–30.0)	
High school or equivalent, %	23.3 (20.7–26.1)	22.4 (19.9–25.0)	28.6 (23.5–34.0)	
College graduate or above, %	58.8 (55–62.5)	61.2 (57.3–65.0)	45.6 (40.6–51.0)	
Family income‐to‐poverty ratio				< 0.001
≤1.0	15.4 (13.1–18)	14.9 (12.6–18.0)	18.2 (14.3–23.0)	
1.0–3.0	36.3 (32.8–39.8)	34.9 (31.3–39.0)	43.9 (38.6–49.0)	
>3.0	48.3 (44.2–52.6)	50.2 (45.9–55.0)	38.0 (32.6–44.0)	
Smoking status				< 0.001
Never smoker, %	54.1 (51.4–56.8)	55.2 (52.2–58.0)	48.4 (42.8–54.0)	
Former smoker, %	23.6 (21.3–26)	21.8 (19.5–24.0)	33.1 (27.9–39.0)	
Current smoker, %	22.3 (19.8–25.1)	23.0 (20.1–26.0)	18.6 (14.9–23.0)	
Drinking status				< 0.001
Nondrinker, %	27.5 (23.8–31.7)	24.4 (20.8–28.0)	44.4 (38.0–51.0)	
Low‐to‐moderate drinker, %	9.96 (8.75–11.32)	10.03 (8.86–11.00)	9.57 (6.91–13.0)	
Heavy drinker, %	62.5 (59–65.9)	65.5 (62.1–69.0)	46.0 (39.6–53.0)	
Physical activity				< 0.001
No activity, %	31.6 (29.1–34.1)	28.8 (26.4–31.0)	46.9 (41.5–52.0)	
Moderate activity, %	32.6 (31.1–34.1)	31.5 (30.1–33.0)	38.6 (32.4–45.0)	
Vigorous activity, %	35.9 (33.2–38.6)	39.7 (37.0–43.0)	14.5 (11.6–18.0)	
Prevalent hypertension, %	30.8 (28.1–33.6)	24.6 (22.3–27.0)	64.8 (60.8–69.0)	< 0.001
Prevalent cardiovascular, %	9.06 (7.57–10.8)	5.80 (4.48–7.00)	26.8 (22.4–32.0)	< 0.001
Prevalent cancer, %	9.13 (7.84–10.6)	7.62 (6.48–9.00)	17.3 (13.6–22.0)	< 0.001
ALA (18:3 n3), % in total serum fatty acids	0.68 (0.66–0.7)	0.67 (0.65–0.69)	0.71 (0.68–0.73)	0.011
EPA (20:5 n3), % in total serum fatty acids	0.5 (0.47–0.52)	0.50 (0.47–0.53)	0.50 (0.46–0.53)	< 0.001
DPAn3 (22:5 n3), % in total serum fatty acids	0.41 (0.41–0.42)	0.41 (0.41–0.42)	0.41 (0.39–0.42)	< 0.001
DHA (22:6 n3), % in total serum fatty acids	1.27 (1.22–1.33)	1.27 (1.21–1.33)	1.28 (1.22–1.34)	0.004
MA (14:1 n5), % in total serum fatty acids	0.07 (0.07–0.07)	0.07 (0.07–0.07)	0.07 (0.07–0.08)	0.514
LA (18:2 n6), % in total serum fatty acids	31.58 (31.37–31.79)	32.04 (31.79–32.29)	29.06 (28.59–29.53)	< 0.001
GLA (18:3 n6), % in total serum fatty acids	0.48 (0.47–0.49)	0.48 (0.47–0.49)	0.48 (0.46–0.50)	0.044
DGLA (20:3 n6), % in total serum fatty acids	1.37 (1.35–1.39)	1.38 (1.36–1.40)	1.33 (1.30–1.36)	0.052
AA (20:4 n6), % in total serum fatty acids	7.26 (7.17–7.35)	7.29 (7.21–7.38)	7.09 (6.82–7.36)	0.038
EDA (20:2 n6), % in total serum fatty acids	0.19 (0.19–0.2)	0.19 (0.19–0.20)	0.19 (0.19–0.20)	0.306
DTA (22:4 n6), % in total serum fatty acids	0.23 (0.22–0.23)	0.23 (0.22–0.23)	0.23 (0.22–0.23)	0.082
DPAn6 (22:5 n6), % in total serum fatty acids	0.18 (0.17–0.18)	0.18 (0.17–0.18)	0.17 (0.17–0.18)	0.302
PA (16:1 n7), % in total serum fatty acids	2.05 (2.01–2.1)	2.02 (1.97–2.06)	2.26 (2.18–2.35)	0.006
VA (18:1 n7), % in total serum fatty acids	1.29 (1.27–1.3)	1.28 (1.26–1.29)	1.34 (1.31–1.37)	0.149
OA (18:1 n9), % in total serum fatty acids	18.64 (18.48–18.81)	18.36 (18.20–18.52)	20.23 (19.88–20.58)	< 0.001
EA (20:1 n9), % in total serum fatty acids	0.12 (0.12–0.13)	0.12 (0.12–0.12)	0.13 (0.13–0.14)	< 0.001
NRA (24:1 n9), % in total serum fatty acids	0.07 (0.06–0.07)	0.74 (0.73–0.75)	0.65 (0.62–0.67)	< 0.001

*Note:* All data analyses conducted in the current study were based on estimates with sample weights provided by NHANES.

Abbreviations: AA, Arachidonic acid; ALA, *α*‐Linolenic acid; BMI, body mass index; DTA, Docosatetraenoic acid; DGLA, dihomo‐gamma‐Linolenic acid; DHA, Docosahexaenoic acid; DPAn3, Docosapentaenoic‐3 acid; DPAn6, Docosapentaenoic‐6 acid; EA, Eicosenoic acid; EDA, Eicosadienoic acid; EPA, Eicosapentaenoic acid; GLA, gamma‐Linolenic acid; LA, Linoleic acid; MA, Myristoleicacid; NRA, Nervonic acid; OA, Oleic acid; PA, Palmitoleic acid; VA, cis‐Vaccenic acid.

^a^Values are weighted means (95% CI) or weighted percentages (95% CI).

^b^ *p* value was assessed with general linear models adjusting for age (continuous variables) or *χ*2 test (bivariate relationships) among two groups.

**Figure 1:**
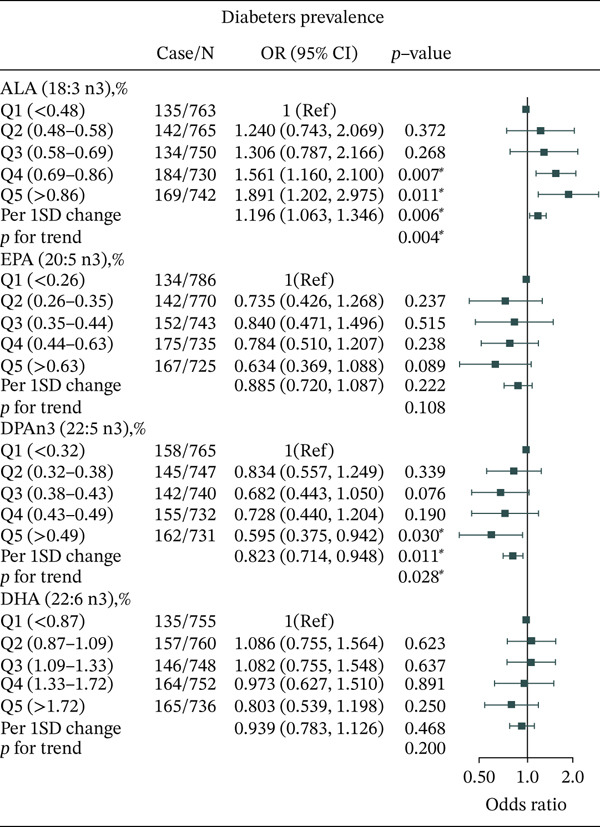
Adjusted ORs for associations between different n‐3 PUFAs and T2DM.

Associations between different n‐3 and n‐6 PUFAs and T2DM prevalence are shown in Tables S18–S21. Among n‐3 PUFAs, ALA (18:3 n3) was positively associated with T2DM in Model 3 (OR = 1.891, 95% CI: 1.202–2.975, *P*_trend_ = 0.004) (Figure 1). Higher DPAn3 (22:5 n3) levels were associated with lower T2DM prevalence (OR = 0.595, 95% CI: 0.375–0.942, *p* = 0.030). No association was observed between EPA (20:5 n3) or DHA (22:6 n3) and T2DM. For n‐5 and n‐7 MUFAs, there was no association between n‐5 or n‐7 MUFAs and T2DM prevalence in Model 3 (Figure S1).

**Figure 2:**
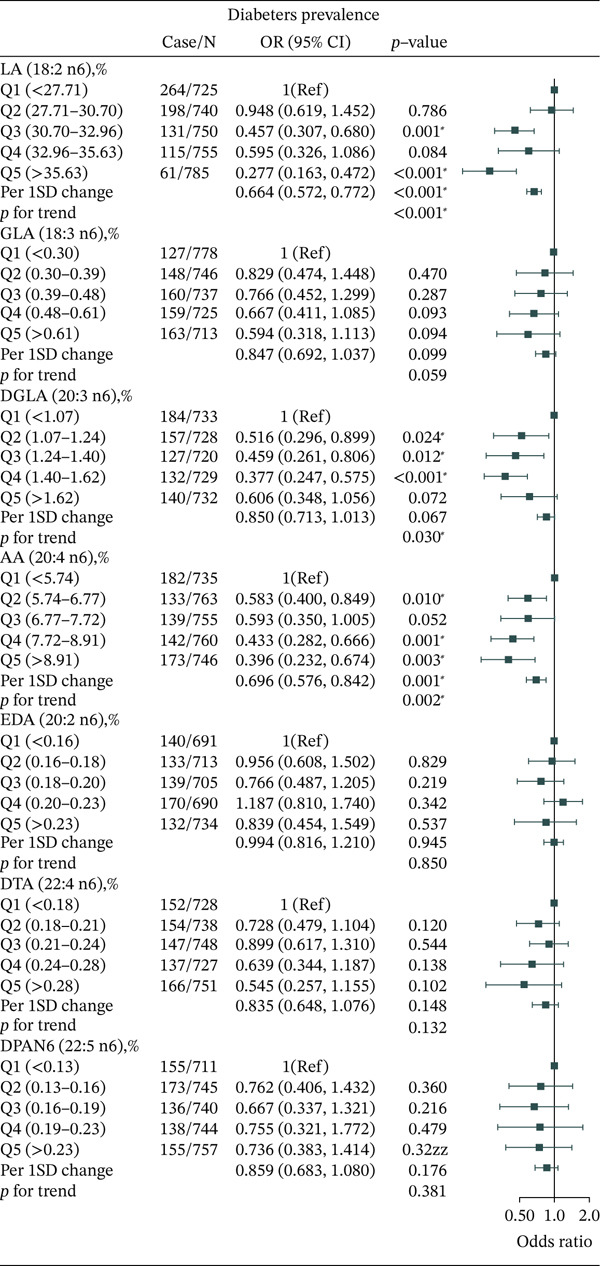
Adjusted OR for associations between different n‐6 PUFAs and T2DM.

With respect to n‐6 PUFAs, several findings were consistent. Compared with the lowest LA (18:2 n6) level (Q1 < 27.71), LA levels in Q3 (30.70–32.96) and Q5 (> 35.63) were associated with lower T2DM prevalence (Q3: OR = 0.457, 95% CI: 0.307–0.680, *p* = 0.001; Q5: OR = 0.277, 95% CI: 0.163–0.472, *p* < 0.014; *P*_trend_ < 0.001). Similar associations were observed for DGLA (20:3 n6) and AA (20:4 n6). However, serum levels of GLA (18:3 n6), EDA (20:2 n6), DTA (22:4 n6), and DPA (22:5 n6) showed no correlation with T2DM (Figure 2).

**Figure 3:**
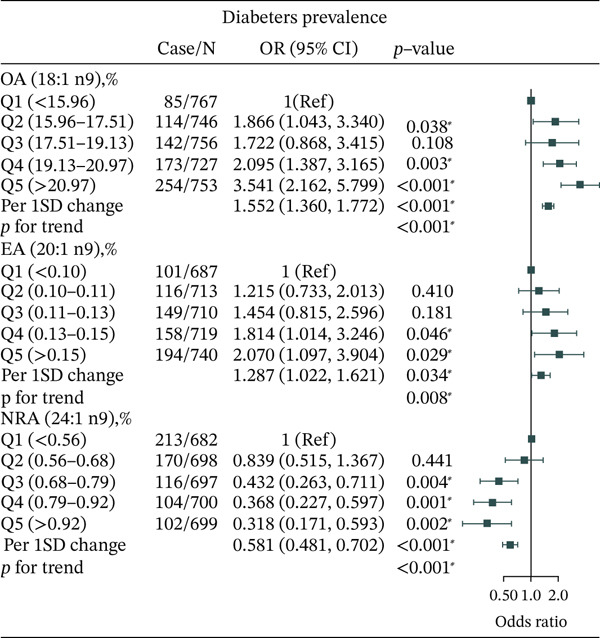
Adjusted OR for associations between different n‐9 PUFAs and T2DM.

Regarding n‐9 MUFAs, OA (18:1 n9) was associated with increased odds of T2DM (OR = 3.541, 95% CI: 2.162–5.799, *p* < 0.001) after adjusting for age, sex, race, education, WC, BMI, PIR, smoking, drinking, physical activity, hypertension, CVD, and cancer (Figure 3). In contrast, NRA (24:1 n9) was associated with lower odds of T2DM (OR = 0.381, 95% CI: 0.171–0.593, *p* = 0.002). EA (20:1 n9) was associated with higher odds of T2DM (OR = 2.070, 95% CI: 1.097–3.904, *p* = 0.029).

**Figure 4:**
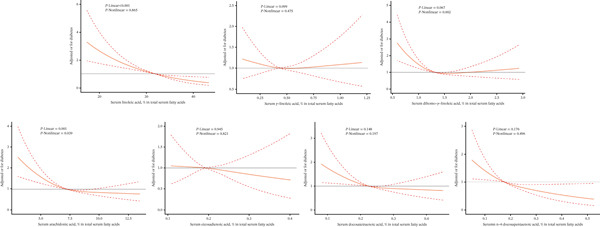
Associations between serum LA (18:2 n6), GLA (18:3 n6), DGLA (20:3 n6), AA (20:4 n6), EDA (20:2 n6), DTA (22:4 n6), and DPAn6 (22:5 n6) with T2DM. Assessed by multivariable‐adjusted ORs using Logistic regression models and restricted cubic splines. The models were adjusted for age (years), sex (male/female), BMI (kg/m^2^), waist circumference (cm), race (non‐Hispanic White/others), education level (less than high school/high school or equivalent/college graduate or above), family income‐to‐poverty ratio (≤ 1.0/1.0–3.0/> 3.0), smoking status (never smoker/former smoker/current smoker), drinking status (nondrinker/low‐to‐moderate drinker/heavy drinker), physical activity (no activity/moderate activity/vigorous activity), hypertension (no/yes), cardiovascular disease (no/yes), and cancer (no/yes). LA, linoleic acid; GLA, gamma‐linolenic acid; DGLA, dihomo‐gamma‐linolenic acid; AA, arachidonic acid; EDA, eicosadienoic acid; DTA, docosatetraenoic acid; DPAn6, n‐6 docosapentaenoic acid.

Nonlinear analyses were also conducted. For patients with T2DM, a linear relationship was observed between ALA (18:3 n3) and DPAn3 (22:5 n3) (*P* − Linear = 0.006; *P* − Linear = 0.011) (Figure S2). For n‐5 MUFAs, PA (16:1 n7) showed a linear association with T2DM (*P* − Linear = 0.023) (Figure S3). DGLA (20:3 n6) and AA (20:4 n6) demonstrated nonlinear associations in T2DM patients (DGLA: *P* − Nonlinear = 0.002; AA: *P* − Nonlinear = 0.039) (Figure 4). EA (20:1 n9) was nonlinearly associated with T2DM (*P* − Nonlinear = 0.023) (Figure S4).

## 4. Discussion

The research findings indicate that n‐3 PUFAs (DPAn3 [22:5 n3]), n‐6 PUFAs (LA [18:2 n6], DGLA [20:3 n6], and AA [20:4 n6]), and n‐9 MUFAs (NRA [24:1 n9]) were associated with lower odds of T2DM, whereas n‐3 PUFAs (ALA [18:3 n3]) and n‐9 MUFAs (EA [20:1 n9] and OA [18:1 n9]) were associated with a higher likelihood of T2DM. In contrast, other n‐3 PUFAs (EPA [20:5 n3] and DHA [22:6 n3]) and n‐6 PUFAs (GLA [18:3 n6], DPA [22:5 n6], DTA [22:4 n6], and EDA [20:2 n6]) showed no associations with T2DM. Additionally, n‐5 and n‐7 MUFAs were not associated with T2DM. The present study also revealed nonlinear associations of serum n‐6 PUFA DGLA (20:3 n6) and n‐9 MUFA EA (20:1 n9) with T2DM. These findings should be interpreted with caution, given the cross‐sectional design. The observed associations do not establish causality and may reflect protective or deleterious effects, or may be secondary to metabolic alterations in established diabetes.

Over recent decades, numerous studies have investigated the role of n‐3 PUFAs in T2DM development, yet results remain controversial. Our study found no association between total serum n‐3 PUFAs (Table S22) and T2DM, consistent with previous findings [18–20]. The relationship between ALA (18:3 n3) and T2DM has been inconsistent. Consistent with our findings, several studies have shown that ALA (18:3 n3) is positively associated with T2DM risk [21, 22]; however, some studies have reported that ALA (18:3 n3) is negatively [23, 24] or not associated with T2DM [25]. These inconsistencies in reporting might be attributed to dietary factors, dietary assessment bias, and sample sizes, or may hinge on the geographical regions [18, 26]. There was no significant association of n‐3 PUFAs (EPA [20:5 n3] and DHA [22:6 n3]) with T2DM in our study, which is consistent with other studies [9, 27, 28]. DPAn3 (22:5n3) is an intermediate metabolite of EPA (20:5n3) and DHA (22:6n3). Compared with DHA (22:6 n3) and EPA (20:5 n3), DPAn3 (22:5 n3) is a less well‐investigated n‐3 PUFA [29]. The mechanism underlying the effect of DPAn3 (22:5 n3) on glycemic control remains unclear [19, 30]. Moreover, few studies have examined the correlation between circulating DPAn3 (22:5 n3) and T2DM. We observed an inverse association between serum DPAn3 (22:5 n3) and T2DM; however, another similar study reported no association [31]. These results indicate that n‐3 PUFAs may play a role in the development of T2DM. However, since our study examined data from a cross‐sectional study, this association could also be interpreted as a consequence of altered metabolic states in established T2DM. In general, the role of n‐3 PUFAs in T2DM still needs further clarity.

In this study, we evaluated eight serum n‐6 PUFAs and their associations with T2DM. Total serum n‐6 PUFAs were inversely associated with T2DM, consistent with previous studies [32, 33]. Among n‐6 PUFAs, AA (20:4 n6) and LA (18:2 n6) are the most extensively studied. LA is the predominant n‐6 PUFA and may benefit glycemic regulation [7, 34]. We found that higher levels of LA were associated with reduced T2DM prevalence, consistent with a meta‐analysis of 20 cohort studies showing lower T2DM risk [35]. Although prospective data suggest a potentially protective effect, our cross‐sectional results may alternatively reflect reduced LA levels secondary to metabolic disruption. AA (20:4 n6), traditionally viewed as proinflammatory [36, 37], has more recently been shown to give rise to anti‐inflammatory metabolites and protect pancreatic *β*‐cells [38–40]. This aligns with our findings and other studies showing reduced T2DM risk with higher AA levels [20, 33] T2DM. However, other studies found no association [41, 42]. Such inconsistencies may reflect differences in populations, analytical approaches, and PUFA quantification methods.

Regarding less widely studied n‐6 PUFA species, including GLA (18:3 n6), DGLA (20:3 n6), EDA (20:2 n6), DTA (22:4 n6), and DPAn6 (22:5 n6), limited evidence exists on their association with T2DM. In the present study, serum DGLA (20:3 n6) and T2DM were inversely associated with T2DM, whereas another study reported a positive association [33]. Our findings suggested no association between GLA (18:3 n6) and T2DM, consistent with similar findings [41]. For DPAn6 and DTA, no significant associations with T2DM were observed. These observations underscore the importance of evaluating these specific n‐6 PUFA subtypes rather than just concentrating on the basic n‐6 PUFAs.

Although n‐3 and n‐6 PUFAs have been extensively studied, MUFAs remain relatively underexplored. In contrast to n‐3 and n‐6 fatty acids, MUFAs are considered “nonessential” [43, 44]. Few studies have evaluated serum n‐5, n‐7, and n‐9 MUFAs. MA (14:1 n5), a representative n‐5 MUFA, has been shown to induce apoptosis in LNCaP cells [45]. Some evidence suggests that n‐7 and n‐9 MUFAs may modulate insulin resistance [11, 46–48]. However, little epidemiological data exist on the relationship between serum MUFAs (n‐5, n‐7, and n‐9) and T2DM. In our study, n‐5 and n‐7 MUFAs were not associated with T2DM, whereas among n‐9 MUFAs, EA (20:1 n9) and OA (18:1 n9) were associated with increased odds of T2DM, and NRA (24:1 n9) was inversely associated. These opposing associations within the n‐9 group suggest complex subtype‐specific functions that may reflect metabolic changes related to T2DM. Given the limited available evidence, further epidemiological investigations are warranted.

A major strength of our study lies in evaluating the associations between objectively measured serum PUFA and MUFA biomarkers and T2DM prevalence using nationally representative NHANES data. We assessed specific n‐3 and n‐6 PUFAs as well as MUFAs (n‐5, n‐7, and n‐9). However, this study also has limitations. First, self‐reported information may introduce misclassification bias, including in sociodemographic variables. Although we adjusted for potential confounders, unmeasured factors such as dietary intake may influence the associations. Second, fatty acid data were available only for 2003–2004 and 2011–2012. In the future, a well‐designed large‐scale population study is needed to determine the relationship between FAs and the prevalence of T2DM. Thirdly, the utility of circulating fatty acid levels in guiding dietary intake may be limited by metabolic conversion and individual differences. Fourth, the single‐time measurement of circulating fatty acids, which may not adequately represent long‐term or stable exposure levels. Finally, due to the observational and cross‐sectional nature of our study, causal inference and temporal direction cannot be determined. Future prospective cohort and longitudinal studies are needed to clarify these associations.

## 5. Conclusions

Overall, this study identified distinct cross‐sectional associations between specific serum fatty acids and T2DM prevalence. Several serum n‐3 and n‐6 PUFAs were inversely associated with T2DM prevalence, whereas n‐9 MUFAs were positively associated with T2DM prevalence, and n‐5 and n‐7 MUFAs showed no association. These findings provide detailed descriptive data and underscore the need for further research to clarify causal relationships linking multiple circulating UFAs with T2DM.

## Author Contributions

Shan Liu and Ying Liu contributed equally to the article. Shan Liu and Ying Liu wrote and revised the manuscript. Lipeng Liu, Fengxia Lv, Huijuan Wang, Xiuyun Zhang, and Shuxia Yue handled the data. Liwen Zhang and Jin Zhou conceived the study and provided supervision. All authors contributed to the article. Shan Liu and Ying Liu contributed equally to this work as co–first authors.

## Funding

This work was supported by the Natural Science Foundation of Hebei Province, No.H2024206065; Medical Science Research Key Project Plan of Hebei Province, No.20181036; Key Research and Development Plan Hebei Province, No.19277795D; Science Research Project of Hebei Education Department, BJ2025204; the Chronic Disease Management Research Project of National Health Commission Capacity Buildingand Continuing Education Center, No.GWJJMB202510024187.

## Disclosure

All authors approved the submitted version.

## Ethics Statement

Ethical review and approval were not required for this study due to the source data of this study being from the NHANES. Informed consent from all participants was not required in the present study according to the national legislation and the institutional requirements.

## Conflicts of Interest

The authors declare no conflict of interest.

## Supporting information


**Supporting information** Additional supporting information can be found online in the Supporting Information section. Includes the baseline characteristics according to quintiles of serum PUFAs and MUFAs (Table S1–Table S17), adjusted OR for associations of PUFAs and MUFAs with the prevalence of T2DM (Table S18–Table S22). Adjusted OR for associations of different n‐5 and n‐7 PUFAs with the prevalence of T2DM (Figure S1), associations of n‐3 PUFAs with the prevalence of T2DM (Figure S2), associations of serum MA (14:1 n5), PA (16:1 n7), and VA (18:1 n7) with the prevalence of T2DM (Figure S3), associations of n‐9 MUFAs with the prevalence of T2DM (Figure S4).

## Data Availability

All data used and analyzed in this study were provided by the National Health and Nutrition Examination Survey (NHANES) and could be downloaded from http://www.cdc.gov/nchs/nhanes/.

## References

[1] American Diabetes Association , 2. Classification and Diagnosis of Diabetes:Standards of Medical Care in Diabetes-2018, Diabetes Care. (2018) 41, no. Supplement_1, S13–S27, 10.2337/dc18-S002, 2-s2.0-85039703274. 29222373

[2] da Rocha F. J. , Ogurtsova K. , Linnenkamp U. , Guariguata L. , Seuring T. , Zhang P. , Cavan D. , and Makaroff L. E. , IDF Diabetes Atlas Estimates of 2014 Global Health Expenditures on Diabetes, Diabetes Research and Clinical Practice. (2016) 117, 48–54, 10.1016/j.diabres.2016.04.016, 2-s2.0-84968877365. 27329022

[3] Imamura F. , Micha R. , Wu J. H. , de Oliveira Otto M. C. , Otite F. O. , Abioye A. I. , and Mozaffarian D. , Effects of Saturated Fat, Polyunsaturated Fat, Monounsaturated Fat, and Carbohydrate on Glucose-Insulin Homeostasis: A Systematic Review and Meta-Analysis of Randomised Controlled Feeding Trials, PLoS Medicine. (2016) 13, no. 7, e1002087, 10.1371/journal.pmed.1002087, 2-s2.0-84979640426, 27434027. 27434027 PMC4951141

[4] Zhuang P. , Liu X. , Li Y. , Li H. , Zhang L. , Wan X. , Wu Y. , Zhang Y. , and Jiao J. , Circulating Fatty Acids and Genetic Predisposition to Type 2 Diabetes: Gene-Nutrient Interaction Analysis, Diabetes Care. (2022) 45, no. 3, 564–575, 10.2337/dc21-2048, 35089324. 35089324

[5] Brown T. J. , Brainard J. , Song F. , Wang X. , Abdelhamid A. , and Hooper L. , Omega-3, Omega-6, and Total Dietary Polyunsaturated Fat for Prevention and Treatment of Type 2 Diabetes Mellitus: Systematic Review and Meta-Analysis of Randomised Controlled Trials, British Medical Journal. (2019) 366, l4697, 10.1136/bmj.l4697, 2-s2.0-85069866781. 31434641 PMC6699594

[6] Ulven S. M. , Leder L. , Elind E. , Ottestad I. , Christensen J. J. , Telle-Hansen V. H. , Skjetne A. J. , Raael E. , Sheikh N. A. , Holck M. , Torvik K. , Lamglait A. , Thyholt K. , Byfuglien M. G. , Granlund L. , Andersen L. F. , and Holven K. B. , Exchanging a Few Commercial, Regularly Consumed Food Items With Improved Fat Quality Reduces Total Cholesterol and LDL-Cholesterol: A Double-Blind, Randomised Controlled Trial, British Journal of Nutrition. (2016) 116, no. 8, 1383–1393, 27737722, 10.1017/S0007114516003445, 2-s2.0-84991224773. 27737722

[7] Telle-Hansen V. H. , Gaundal L. , and Myhrstad M. C. W. , Polyunsaturated Fatty Acids and Glycemic Control in Type 2 Diabetes, Nutrients. (2019) 11, no. 5, 10.3390/nu11051067, 2-s2.0-85066833972. PMC6566834 31091649

[8] Meyer K. A. , Kushi L. H. , Jacobs D. R. , and Folsom A. R. , Dietary Fat and Incidence of Type 2 Diabetes in Older Iowa Women, Diabetes Care. (2001) 24, no. 9, 1528–1535, 11522694, 10.2337/diacare.24.9.1528, 2-s2.0-0035463894. 11522694

[9] Forouhi N. G. , Imamura F. , Sharp S. J. , Koulman A. , Schulze M. B. , Zheng J. , Ye Z. , Sluijs I. , Guevara M. , Huerta J. M. , Kröger J. , Wang L. Y. , Summerhill K. , Griffin J. L. , Feskens E. J. , Affret A. , Amiano P. , Boeing H. , Dow C. , Fagherazzi G. , Franks P. W. , Gonzalez C. , Kaaks R. , Key T. J. , Khaw K. T. , Kühn T. , Mortensen L. M. , Nilsson P. M. , Overvad K. , Pala V. , Palli D. , Panico S. , Quirós J. R. , Rodriguez-Barranco M. , Rolandsson O. , Sacerdote C. , Scalbert A. , Slimani N. , Spijkerman A. M. , Tjonneland A. , Tormo M. J. , Tumino R. , van der A. D. , van der Schouw Y. , Langenberg C. , Riboli E. , and Wareham N. J. , Association of Plasma Phospholipid N-3 and N-6 Polyunsaturated Fatty Acids With Type 2 Diabetes: The EPIC-InterAct Case-Cohort Study, PLoS Medicine. (2016) 13, no. 7, e1002094, 10.1371/journal.pmed.1002094, 2-s2.0-84980332028, 27434045. 27434045 PMC4951144

[10] Zhou Y. , Tian C. , and Jia C. , Association of Fish and N-3 Fatty Acid Intake With the Risk of Type 2 Diabetes: A Meta-Analysis of Prospective Studies, British Journal of Nutrition. (2012) 108, no. 3, 408–417, 10.1017/S0007114512002036, 2-s2.0-84865255083, 22857650. 22857650

[11] Medeiros-de-Moraes I. M. , Gonçalves-de-Albuquerque C. F. , Kurz A. R. M. , Oliveira F. M. J. , de Abreu V. H. P. , Torres R. C. , Carvalho V. F. , Estato V. , Bozza P. T. , Sperandio M. , de Castro-Faria-Neto H. C. , and Silva A. R. , Omega-9 Oleic Acid, the Main Compound of Olive Oil, Mitigates Inflammation During Experimental Sepsis, Oxidative Medicine and Cellular Longevity. (2018) 2018, 6053492, 10.1155/2018/6053492, 2-s2.0-85058609006. 30538802 PMC6260523

[12] Levy B. D. , Clish C. B. , Schmidt B. , Gronert K. , and Serhan C. N. , Lipid Mediator Class Switching During Acute Inflammation: Signals in Resolution, Nature Immunology. (2001) 2, no. 7, 612–619, 10.1038/89759, 2-s2.0-0034916569, 11429545. 11429545

[13] Bolsoni-Lopes A. , Festuccia W. T. , Chimin P. , Farias T. S. , Torres-Leal F. L. , Cruz M. M. , Andrade P. B. , Hirabara S. M. , Lima F. B. , and Alonso-Vale M. I. , Palmitoleic Acid (N-7) Increases White Adipocytes GLUT4 Content and Glucose Uptake in Association With AMPK Activation, Lipids in Health and Disease. (2014) 13, no. 1, 10.1186/1476-511X-13-199, 2-s2.0-84925273406. PMC4364637 25528561

[14] Hodson L. , Skeaff C. M. , and Fielding B. A. , Fatty Acid Composition of Adipose Tissue and Blood in Humans and Its Use as a Biomarker of Dietary Intake, Progress in Lipid Research. (2008) 47, no. 5, 348–380, 10.1016/j.plipres.2008.03.003, 2-s2.0-44949160947, 18435934. 18435934

[15] Panezai J. and van Dyke T. , Polyunsaturated Fatty Acids and Their Immunomodulatory Actions in Periodontal Disease, Nutrients. (2023) 15, no. 4, 10.3390/nu15040821. PMC9965392 36839179

[16] Qian F. , Ardisson Korat A. V. , Imamura F. , Marklund M. , Tintle N. , Virtanen J. K. , Zhou X. , Bassett J. K. , Lai H. , Hirakawa Y. , Chien K. L. , Wood A. C. , Lankinen M. , Murphy R. A. , Samieri C. , Pertiwi K. , de Mello V. D. , Guan W. , Forouhi N. G. , Wareham N. , Hu I. C. F. B. , Riserus U. , Lind L. , Harris W. S. , Shadyab A. H. , Robinson J. G. , Steffen L. M. , Hodge A. , Giles G. G. , Ninomiya T. , Uusitupa M. , Tuomilehto J. , Lindström J. , Laakso M. , Siscovick D. S. , Helmer C. , Geleijnse J. M. , Wu J. H. Y. , Fretts A. , Lemaitre R. N. , Micha R. , Mozaffarian D. , Sun Q. , and Fatty Acids and Outcomes Research Consortium (FORCE) , N-3 Fatty Acid Biomarkers and Incident Type 2 Diabetes: An Individual Participant-Level Pooling Project of 20 Prospective Cohort Studies, Diabetes Care. (2021) 44, no. 5, 1133–1142, 10.2337/dc20-2426, 33658295. 33658295 PMC8132316

[17] Zhang L. , Liu J. , Cao Y. , Liu S. , Zhao W. , Wang C. , Banzhao S. , Liu Z. , and Liu L. , Association Between Circulating Levels of Unsaturated Fatty Acids and Risk for Prediabetes in the NHANES 2003-2004 and 2011-2012, Diabetes Research and Clinical Practice. (2024) 213, 111728, 10.1016/j.diabres.2024.111728. 38838943

[18] Chen C. , Yang Y. , Yu X. , Hu S. , and Shao S. , Association Between Omega-3 Fatty Acids Consumption and the Risk of Type 2 Diabetes: A Meta-Analysis of Cohort Studies, Journal of Diabetes Investigation. (2017) 8, no. 4, 480–488, 10.1111/jdi.12614, 2-s2.0-85011655865, 28032469. 28032469 PMC5497038

[19] Mahendran Y. , Ågren J. , Uusitupa M. , Cederberg H. , Vangipurapu J. , Stančáková A. , Schwab U. , Kuusisto J. , and Laakso M. , Association of Erythrocyte Membrane Fatty Acids With Changes in Glycemia and Risk of Type 2 Diabetes, American Journal of Clinical Nutrition. (2014) 99, no. 1, 79–85, 10.3945/ajcn.113.069740, 2-s2.0-84891462249, 24153340. 24153340

[20] Li Y. , Shen H. , Li Y. , Bi M. , Bi Y. , Che X. , Tian S. , and Liu Y. , Sex-Specific Differences in the Associations Between Omega-6 Polyunsaturated Fatty Acids and Type 2 Diabetes in Chinese People, Frontiers in Nutrition. (2021) 8, 739850, 10.3389/fnut.2021.739850. 34746208 PMC8568790

[21] Alhazmi A. , Stojanovski E. , Garg M. L. , and McEvoy M. , Fasting Whole Blood Fatty Acid Profile and Risk of Type 2 Diabetes in Adults: A Nested Case Control Study, PLoS One. (2014) 9, no. 5, e97001, 10.1371/journal.pone.0097001, 2-s2.0-84901194589, 24816459. 24816459 PMC4016225

[22] Hodge A. M. , English D. R. , O′Dea K. , Sinclair A. J. , Makrides M. , Gibson R. A. , and Giles G. G. , Plasma Phospholipid and Dietary Fatty Acids as Predictors of Type 2 Diabetes: Interpreting the Role of Linoleic Acid, American Journal of Clinical Nutrition. (2007) 86, no. 1, 189–197, 10.1093/ajcn/86.1.189, 17616780. 17616780

[23] Djoussé L. , Biggs M. L. , Lemaitre R. N. , King I. B. , Song X. , Ix J. H. , Mukamal K. J. , Siscovick D. S. , and Mozaffarian D. , Plasma Omega-3 Fatty Acids and Incident Diabetes in Older Adults, American Journal of Clinical Nutrition. (2011) 94, no. 2, 527–533, 10.3945/ajcn.111.013334, 2-s2.0-79960867594, 21593500. 21593500 PMC3142727

[24] Wang L. , Folsom A. R. , Zheng Z. J. , Pankow J. S. , and Eckfeldt J. H. , Plasma Fatty Acid Composition and Incidence of Diabetes in Middle-Aged Adults: The Atherosclerosis Risk in Communities (ARIC) Study, American Journal of Clinical Nutrition. (2003) 78, no. 1, 91–98, 10.1093/ajcn/78.1.91, 12816776. 12816776

[25] Qian F. , Ardisson Korat A. V. , Imamura F. , Marklund M. , Tintle N. , Virtanen J. K. , Zhou X. , Bassett J. K. , Lai H. , Hirakawa Y. , Chien K. L. , Wood A. C. , Lankinen M. , Murphy R. A. , Samieri C. , Pertiwi K. , de Mello V. D. , Guan W. , Forouhi N. G. , Wareham N. , Hu I. , Riserus U. , Lind L. , Harris W. S. , Shadyab A. H. , Robinson J. G. , Steffen L. M. , Hodge A. , Giles G. G. , Ninomiya T. , Uusitupa M. , Tuomilehto J. , Lindström J. , Laakso M. , Siscovick D. S. , Helmer C. , Geleijnse J. M. , Wu J. H. Y. , Fretts A. , Lemaitre R. N. , Micha R. , Mozaffarian D. , Sun Q. , and Fatty Acids and Outcomes Research Consortium (FORCE) , N-3 Fatty Acid Biomarkers and Incident Type 2 Diabetes: An Individual Participant-Level Pooling Project of 20 Prospective Cohort Studies, Diabetes Care. (2021) 44, no. 5, 1133–1142, 10.2337/dc20-2426, 33658295. 33658295 PMC8132316

[26] Wallin A. , Di Giuseppe D. , Orsini N. , Patel P. S. , Forouhi N. G. , and Wolk A. , Fish Consumption, Dietary Long-Chain N-3 Fatty Acids, and Risk of Type 2 Diabetes: Systematic Review and Meta-Analysis of Prospective Studies, Diabetes Care. (2012) 35, no. 4, 918–929, 10.2337/dc11-1631, 2-s2.0-84862067335, 22442397. 22442397 PMC3308304

[27] Xun P. and He K. , Fish Consumption and Incidence of Diabetes: Meta-Analysis of Data From 438,000 Individuals in 12 Independent Prospective Cohorts With an Average 11-Year Follow-Up, Diabetes Care. (2012) 35, no. 4, 930–938, 10.2337/dc11-1869, 2-s2.0-84862099934, 22442398. 22442398 PMC3308299

[28] Lim G. B. , No Benefit of N-3 Fatty Acid Supplements in Diabetes, Nature Reviews Cardiology. (2018) 15, no. 11, 10.1038/s41569-018-0090-0, 2-s2.0-85053438551, 30214020. 30214020

[29] Zheng J. S. , Lin J. S. , Dong H. L. , Zeng F. F. , Li D. , Song Y. , and Chen Y. M. , Association of Erythrocyte N-3 Polyunsaturated Fatty Acids With Incident Type 2 Diabetes in a Chinese Population, Clinical Nutrition. (2019) 38, no. 5, 2195–2201, 10.1016/j.clnu.2018.09.018, 2-s2.0-85054430552, 30309708. 30309708

[30] Lankinen M. A. , Stančáková A. , Uusitupa M. , Ågren J. , Pihlajamäki J. , Kuusisto J. , Schwab U. , and Laakso M. , Plasma Fatty Acids as Predictors of Glycaemia and Type 2 Diabetes, Diabetologia. (2015) 58, no. 11, 2533–2544, 10.1007/s00125-015-3730-5, 2-s2.0-84942991526, 26277381. 26277381

[31] Virtanen J. K. , Mursu J. , Voutilainen S. , Uusitupa M. , and Tuomainen T. P. , Serum Omega-3 Polyunsaturated Fatty Acids and Risk of Incident Type 2 Diabetes in Men: The Kuopio Ischemic Heart Disease Risk Factor Study, Diabetes Care. (2014) 37, no. 1, 189–196, 10.2337/dc13-1504, 2-s2.0-84892419206, 24026545. 24026545

[32] Zhuang P. , Liu X. , Li Y. , Li H. , Zhang L. , Wan X. , Wu Y. , Zhang Y. , and Jiao J. , Circulating Fatty Acids and Genetic Predisposition to Type 2 Diabetes: Gene-Nutrient Interaction Analysis, Diabetes Care. (2022) 45, no. 3, 564–575, 10.2337/dc21-2048, 35089324. 35089324

[33] Yary T. , Voutilainen S. , Tuomainen T. P. , Ruusunen A. , Nurmi T. , and Virtanen J. K. , Serum N-6 Polyunsaturated Fatty Acids, *Δ*5- and *Δ*6-Desaturase Activities, and Risk of Incident Type 2 Diabetes in Men: The Kuopio Ischaemic Heart Disease Risk Factor Study, American Journal of Clinical Nutrition. (2016) 103, no. 5, 1337–1343, 10.3945/ajcn.115.128629, 2-s2.0-84969540978, 27009754. 27009754

[34] Belury M. A. , Cole R. M. , Snoke D. B. , Banh T. , and Angelotti A. , Linoleic Acid, Glycemic Control and Type 2 Diabetes, Prostaglandins, Leukotrienes, and Essential Fatty Acids. (2018) 132, 30–33, 10.1016/j.plefa.2018.03.001, 2-s2.0-85051640538, 29735020. 29735020 PMC11190750

[35] Wu J. H. Y. , Marklund M. , Imamura F. , Tintle N. , Ardisson Korat A. V. , de Goede J. , Zhou X. , Yang W. S. , de Oliveira Otto M. C. , Kröger J. , Qureshi W. , Virtanen J. K. , Bassett J. K. , Frazier-Wood A. C. , Lankinen M. , Murphy R. A. , Rajaobelina K. , Del Gobbo L. C. , Forouhi N. G. , Luben R. , Khaw K. T. , Wareham N. , Kalsbeek A. , Veenstra J. , Luo J. , Hu F. B. , Lin H. J. , Siscovick D. S. , Boeing H. , Chen T. A. , Steffen B. , Steffen L. M. , Hodge A. , Eriksdottir G. , Smith A. V. , Gudnason V. , Harris T. B. , Brouwer I. A. , Berr C. , Helmer C. , Samieri C. , Laakso M. , Tsai M. Y. , Giles G. G. , Nurmi T. , Wagenknecht L. , Schulze M. B. , Lemaitre R. N. , Chien K. L. , Soedamah-Muthu S. S. , Geleijnse J. M. , Sun Q. , Harris W. S. , Lind L. , Ärnlöv J. , Riserus U. , Micha R. , and Mozaffarian D. , Omega-6 Fatty Acid Biomarkers and Incident Type 2 Diabetes: Pooled Analysis of Individual-Level Data for 39?740 Adults From 20 Prospective Cohort Studies, Lancet Diabetes and Endocrinology. (2017) 5, no. 12, 965–974, 10.1016/S2213-8587(17)30307-8, 2-s2.0-85031309422, 29032079. 29032079 PMC6029721

[36] Patterson E. , Wall R. , Fitzgerald G. F. , Ross R. P. , and Stanton C. , Health Implications of High Dietary Omega-6 Polyunsaturated Fatty Acids, Journal of Nutrition and Metabolism. (2012) 2012, 539426, 10.1155/2012/539426, 2-s2.0-84870579279. 22570770 PMC3335257

[37] Fritsche K. L. , The Science of Fatty Acids and Inflammation, Advances in Nutrition. (2015) 6, no. 3, 293s–301s, 10.3945/an.114.006940, 2-s2.0-84943660984, 25979502. 25979502 PMC4424767

[38] Harris W. S. and Shearer G. C. , Omega-6 Fatty Acids and Cardiovascular Disease: Friend, Not Foe?, Circulation. (2014) 130, no. 18, 1562–1564, 10.1161/CIRCULATIONAHA.114.012534, 2-s2.0-84922394106, 25161044. 25161044

[39] Suresh Y. and Das U. N. , Protective Action of Arachidonic Acid Against Alloxan-Induced Cytotoxicity and Diabetes Mellitus, Prostaglandins, Leukotrienes, and Essential Fatty Acids. (2001) 64, no. 1, 37–52, 10.1054/plef.2000.0236, 2-s2.0-0035126282, 11161584. 11161584

[40] Krishna Mohan I. and Das U. N. , Prevention of Chemically Induced Diabetes Mellitus in Experimental Animals by Polyunsaturated Fatty Acids, Nutrition. (2001) 17, no. 2, 126–151, 10.1016/S0899-9007(00)00468-8, 2-s2.0-0035113112, 11240341. 11240341

[41] Zhu X. , Chen L. , Lin J. , Ba M. , Liao J. , Zhang P. , and Zhao C. , Association Between Fatty Acids and the Risk of Impaired Glucose Tolerance and Type 2 Diabetes Mellitus in American Adults: NHANES 2005-2016, Nutrition & Diabetes. (2023) 13, no. 1, 10.1038/s41387-023-00236-4, 37127641. PMC10151340 37127641

[42] Prada M. , Eichelmann F. , Wittenbecher C. , Kuxhaus O. , and Schulze M. B. , Plasma Lipidomic N-6 Polyunsaturated Fatty Acids and Type 2 Diabetes Risk in the EPIC-Potsdam Prospective Cohort Study, Diabetes Care. (2023) 46, no. 4, 836–844, 10.2337/dc22-1435, 36787959. 36787959 PMC10090908

[43] De Lorgeril M. , Essential Polyunsaturated Fatty Acids, Inflammation, Atherosclerosis and Cardiovascular Diseases, Subcellular Biochemistry. (2007) 42, 283–297, 17612056. 17612056 10.1007/1-4020-5688-5_13

[44] Paton C. M. and Ntambi J. M. , Biochemical and Physiological Function of Stearoyl-CoA Desaturase, American Journal of Physiology - Endocrinology and Metabolism. (2009) 297, no. 1, E28–E37, 10.1152/ajpendo.90897.2008, 2-s2.0-67650088283, 19066317. 19066317 PMC2711665

[45] Iguchi K. , Okumura N. , Usui S. , Sajiki H. , Hirota K. , and Hirano K. , Myristoleic Acid, a Cytotoxic Component in the Extract From Serenoa Repens, Induces Apoptosis and Necrosis in Human Prostatic LNCaP Cells, Prostate. (2001) 47, no. 1, 59–65, 10.1002/pros.1047.abs, 11304730. 11304730

[46] Souza C. O. , Teixeira A. A. , Lima E. A. , Batatinha H. A. , Gomes L. M. , Carvalho-Silva M. , Mota I. T. , Streck E. L. , Hirabara S. M. , and Rosa Neto J. C. , Palmitoleic Acid (N-7) Attenuates the Immunometabolic Disturbances Caused by a High-Fat Diet Independently of PPAR*α* , Mediators of Inflammation. (2014) 2014, 582197, 10.1155/2014/582197, 2-s2.0-84929112616. 25147439 PMC4131426

[47] Nunes E. A. and Rafacho A. , Implications of Palmitoleic Acid (Palmitoleate) on Glucose Homeostasis, Insulin Resistance and Diabetes, Current Drug Targets. (2017) 18, no. 6, 619–628, 10.2174/1389450117666151209120345, 2-s2.0-85019610398, 26648072. 26648072

[48] Farag M. A. and Gad M. Z. , Omega-9 Fatty Acids: Potential Roles in Inflammation and Cancer Management, Journal of Genetic Engineering and Biotechnology. (2022) 20, no. 1, 10.1186/s43141-022-00329-0, 35294666. PMC8927560 35294666

