# Supplementary material for: Relationship Between Serum Levels of Unsaturated Fatty Acids and Type 2 Diabetes Mellitus: A Cross‐Sectional Analysis of NHANES 2003–2004 and 2011–2012
Source: J Diabetes Res. 2026 Mar 1;2026:1153035. doi: 10.1155/jdr/1153035 (PMC12950622; doi:10.1155/jdr/1153035)

**Relationship between serum levels of unsaturated fatty acids and type 2 diabetes mellitus: A cross-sectional analysis of NHANES 2003–2004 and 2011–2012**

Shan Liu^1*^, Ying Liu^2*^, Lipeng Liu^3,4^, Fengxia Lv^4^, Huijuan Wang^1^, Xiuyun Zhang^1^, Shuxia Yue^1^, Liwen Zhang^5#^, Jin Zhou^6#^

^1^Department of Endocrinology, The Second Hospital of Shijiazhuang, Shijiazhuang, Hebei, 050051, China.

^2^Obstetrical Department Ⅷ, The Fourth Hospital of Shijiazhuang, Shijiazhuang, Hebei, 050011, China.

^3^Hebei Provincial Key Laboratory of Basic Medicine for Diabetes, The Second Hospital of Shijiazhuang, Shijiazhuang, Hebei, 050051, China

^4^College of Veterinary, Hebei Agricultural University, Baoding, 071000, China.

^5^Department of Epidemiology and Statistics, School of Public Health, Hebei Medical University, Hebei Key Laboratory of Environment and Human Health, Shijiazhuang, Hebei, 050017, China.

^6^Health Care Center, The First Hospital of Hebei Medical University, Shijiazhuang, Hebei, 050000, China.

^*^Shan Liu and Ying Liu contributed equally to this study and share first authorship.

**Correspondence:**

Jin Zhou, Health Care Center, the First Hospital of Hebei Medical University, Shijiazhuang, Hebei, 050000, China.

E-mail: [57101298@hebmu.edu.cn](mailto:57101298@hebmu.edu.cn).

Liwen Zhang, Department of Epidemiology and Statistics, School of Public Health, Hebei Medical University, Hebei Key Laboratory of Environment and Human Health, Shijiazhuang, Hebei, 050017, China.

E-mail: [19001648@hebmu.edu.cn](mailto:19001648@hebmu.edu.cn).

**Supplemental Figure Legends**

**Figure S1.** Adjusted OR for associations of different n-5 and n-7 PUFAs with the prevalence of T2DM

**Figure S2.** Associations of serum ALA (18:3 n3), EPA (20:5 n3), DPAn3 (22:5 n3) and DHA (22:6 n3) with the prevalence of T2DM. Assessed by multivariable-adjusted ORs using of Logistic regression models and restricted cubic splines. The models were adjusted for age (years), sex (male/female), BMI (kg/m^2^), Waist circumference (cm), race (non-Hispanic white/others), education level (Less than high school/High school or equivalent/College graduate or above), family income-to-poverty ratio (≤1.0/1.0-3.0/>3.0), smoking status(never smoker/former smoker/current smoker), drinking status (Nondrinker/Low-to-moderate drinker/Heavy drinker), Physical activity (No activity/Moderate activity/Vigorous activity), hypertension (no/yes), cardiovascular disease (no/yes) and cancer (no/yes); ALA: a-Linolenic acid; EPA: eicosapenta- enoic acid; DPAn3: n-3 Docosapentaenoic acid; DHA: docosahexaenoic acid.

**Figure S3.** Associations of serum MA (14:1 n5), PA (16:1 n7) and VA (18:1 n7) with the prevalence of T2DM. Assessed by multivariable-adjusted ORs using of Logistic regression models and restricted cubic splines. The models were adjusted for age (years), sex (male/female), BMI (kg/m^2^), Waist circumference (cm), race (non-Hispanic white/others), education level (Less than high school/High school or equivalent/College graduate or above), family income-to-poverty ratio (≤1.0/1.0-3.0/>3.0), smoking status(never smoker/former smoker/current smoker), drinking status (Nondrinker/Low-to-moderate drinker/Heavy drinker), Physical activity (No activity/Moderate activity/Vigorous activity), hypertension (no/yes), cardiovascular disease (no/yes) and cancer (no/yes); MA: myristic acid; PA: palmitoleic acid; VA: cis-Vaccenic acid.

**Figure S4.** Associations of serum OA (18:1 n9), EA (20:1 n9) and NRA (24:1 n9) with the prevalence of T2DM. Assessed by multivariable-adjusted ORs using of Logistic regression models and restricted cubic splines. The models were adjusted for age (years), sex (male/female), BMI (kg/m^2^), Waist circumference (cm), race (non-Hispanic white/others), education level (Less than high school/High school or equivalent/College graduate or above), family income-to-poverty ratio (≤1.0/1.0-3.0/>3.0), smoking status(never smoker/former smoker/current smoker), drinking status (Nondrinker/Low-to-moderate drinker/Heavy drinker), Physical activity (No activity/Moderate activity/Vigorous activity), hypertension (no/yes), cardiovascular disease (no/yes) and cancer (no/yes); OA: Oleic acid; EA: Eicosenoic acid; NRA: Nervonic acid.

**SUPPLEMENTAL TABLES**

Table S1. Baseline characteristics according to quintiles of serum α-Linolenic acid (ALA)^‡^

|  | Serum α-Linolenic acid quintile (%) | | | | | *p*-trend |
| --- | --- | --- | --- | --- | --- | --- |
|  | Q1(<0.48) | Q2(0.48-0.58) | Q3(0.58-0.69) | Q4(0.69-0.86) | Q5(>0.86) |  |
| Age, years | 45.2(43.7-46.8) | 45.2(43.7-46.8) | 45.2(43.7-46.8) | 45.2(43.7-46.8) | 45.2(43.7-46.8) | 0.696 |
| Male, % | 51.2(46.9-55.5) | 48.2(43.4-53.1) | 43.7(39.0-48.4) | 42.3(37.6-47.2) | 53.8(48.3-59.2) | 0.008 |
| Non-Hispanic white | 71.5(64.4-77.7) | 71.7(65.4-77.3) | 70.0(64.5-75.0) | 71.0(63.0-77.9) | 62.3(55.2-69.0) | 0.014 |
| BMI, kg/m^2^ | 28.5(27.8-29.2) | 28.1(27.4-28.7) | 28.6(27.9-29.4) | 28.2(27.6-28.9) | 28.1(27.3-28.8) | 0.542 |
| WAIST, cm | 97.8(96.1-99.4) | 96.6(94.9-98.4) | 97.9(96-99.9) | 97.3(95.4-99.2) | 97.9(95.5-100.3) | 0.734 |
| Education |  |  |  |  |  | 0.055 |
| Less than high school, % | 18.0(14.2-22.6) | 18.6(15.1-22.6) | 14.8(11.5-18.7) | 16.5(13.5-20.0) | 21.7(16.9-27.4) |  |
| High school or equivalent, % | 23.9(19.5-29.1) | 27.1(22.2-32.6) | 23.5(19.4-28.2) | 21.9(18.0-26.3) | 19.9(16.4-24.0) |  |
| College graduate or above, % | 58.1(52.2-63.7) | 54.3(48.3-60.2) | 61.7(56.7-66.6) | 61.6(55.5-67.4) | 58.4(52.0-64.4) |  |
| Family income-to-poverty ratio |  |  |  |  |  | 0.728 |
| ≤1.0 | 16.1(12.6-20.4) | 15.7(12.1-20.2) | 12.7(10.4-15.5) | 15.9(12.0-20.8) | 16.4(12.9-20.7) |  |
| 1.0-3.0 | 37.0(31.5-42.8) | 36.8(32.1-41.7) | 35.8(31.0-40.9) | 37.2(30.5-44.3) | 34.5(29.2-40.1) |  |
| >3.0 | 46.9(41.3-52.6) | 47.5(41.1-53.9) | 51.5(46.3-56.7) | 46.9(39.3-54.6) | 49.1(42.8-55.5) |  |
| Smoking status |  |  |  |  |  | <0.001 |
| Never smoker, % | 46.7(42.3-51.1) | 51.7(48.3-55.0) | 55.4(50.1-60.6) | 55.2(49.5-60.8) | 62.3(57.4-66.9) |  |
| Former smoker, % | 23.1(19.1-27.7) | 24.0(20.6-27.9) | 26.9(22.3-32.0) | 24.8(21.1-28.8) | 18.8(15.4-22.7) |  |
| Current smoker, % | 30.2(26.1-34.6) | 24.3(20.1-28.9) | 17.7(14.4-21.6) | 20.1(16.1-24.8) | 19.0(14.1-25.0) |  |
| Drinking status |  |  |  |  |  | 0.070 |
| Nondrinker, % | 27.3(21.9-33.4) | 23.7(19.0-29.2) | 27.2(22.8-32.0) | 29.4(23.5-36.0) | 30.8(24.9-37.4) |  |
| Low-to-moderate drinker, % | 9.3(7.2-12.1) | 9.6(6.9-13.1) | 9.8(7.4-12.9) | 7.8(5.6-10.9) | 13.5(10.4-17.4) |  |
| Heavy drinker, % | 63.4(58.1-68.4) | 66.7(62.0-71.1) | 63.0(57.8-68.0) | 62.8(55.8-69.3) | 55.7(49.8-61.5) |  |
| Physical activity |  |  |  |  |  | 0.426 |
| No activity, % | 34.5(29.2-40.2) | 32.6(28.4-37.0) | 29.7(25.6-34.0) | 30.9(26.1-36.3) | 30.1(25.4-35.1) |  |
| Moderate activity, % | 31.9(28.4-35.6) | 31.5(27.9-35.4) | 34.1(29.9-38.7) | 35.7(30.5-41.3) | 29.6(24.6-35.2) |  |
| Vigorous activity, % | 33.6(28.9-38.7) | 35.9(32.3-39.7) | 36.2(31.0-41.7) | 33.4(28.6-38.5) | 40.3(33.7-47.3) |  |
| Prevalent hypertension, % | 34.2(30.1-38.5) | 28.6(23.5-34.2) | 28.2(24.5-32.2) | 29.7(25.9-33.7) | 33.0(27.2-39.4) | 0.203 |
| Prevalent cardiovascular, % | 7.5(5.4-10.3) | 9.6(7.2-12.6) | 9.7(6.8-13.5) | 9.4(6.8-12.7) | 9.3(7.1-12.0) | 0.689 |
| Prevalent cancer, % | 10.1(7.2-14.1) | 9.0(6.7-11.8) | 7.6(5.5-10.4) | 10.0(7.4-13.4) | 9.0(6.8-11.8) | 0.605 |

^‡^Values are weighted means (95% CI) or weighted percentages (95% CI).

^*^*P* <0.05. *P* was assessed with General linear models adjusting for age (continuous variables) or χ^2^ test (bivariate relationships).

All data analyses conducted in the current study were based on estimates with sample weights provided by NHANES.

Table S2. Baseline characteristics according to quintiles of serum Eicosapentaenoic acid (EPA)^‡^

|  | Serum Eicosapentaenoic acid quintile (%) | | | | | *p*-trend |
| --- | --- | --- | --- | --- | --- | --- |
|  | Q1(<0.26) | Q2(0.26-0.35) | Q3(0.35-0.44) | Q4(0.44-0.63) | Q5(>0.63) |  |
| Age, years | 39.5(37.6-41.4) | 43.4(42.1-44.8) | 46.1(44.3-47.9) | 48.3(46.6-50.1) | 52.7(50.9-54.4) | <0.001 |
| Male, % | 45.3(41.7-49.0) | 50.1(45.7-54.5) | 46.3(41.3-51.3) | 52.5(48.1-56.9) | 44.5(41.0-48.1) | 0.049 |
| Non-Hispanic white | 58.3(49.9-66.2) | 69.3(62.4-75.5) | 71.4(64.2-77.7) | 74.2(68.5-79.1) | 73.3(67.1-78.7) | <0.001 |
| BMI, kg/m^2^ | 28.1(27.5-28.6) | 28.8(28.1-29.5) | 28.4(28.0-28.9) | 28.9(28.2-29.5) | 27.2(26.6-27.8) | 0.013 |
| WAIST, cm | 96.0(94.3-97.7) | 98.2(96.4-100.0) | 97.4(96.2-98.5) | 100(98.3-101.8) | 95.6(94.0-97.3) | 0.029 |
| Education |  |  |  |  |  | <0.001 |
| Less than high school, % | 24.6(19.4-30.6) | 20.3(17.2-23.9) | 16.1(12.1-21.0) | 17.3(13.8-21.4) | 11.4(8.4-15.2) |  |
| High school or equivalent, % | 23.1(19.5-27.3) | 26.1(21.9-30.7) | 23.1(18.7-28) | 25.7(19.2-33.5) | 18.1(14.3-22.8) |  |
| College graduate or above, % | 52.3(46.2-58.3) | 53.6(48.6-58.5) | 60.9(55-66.4) | 57.0(49.4-64.3) | 70.5(64.2-76.1) |  |
| Family income-to-poverty ratio |  |  |  |  |  | <0.001 |
| ≤1.0 | 22.0(18.0-26.7) | 18.0(14.8-21.8) | 14.1(11.3-17.4) | 14.4(10.9-18.6) | 8.7(6.8-11.1) |  |
| 1.0-3.0 | 39.2(35.2-43.4) | 39.5(33.7-45.5) | 37.5(32.3-43.1) | 36.3(29.6-43.6) | 28.6(22.7-35.3) |  |
| >3.0 | 38.8(33.4-44.4) | 42.5(37.5-47.7) | 48.4(42.7-54.1) | 49.3(41.0-57.7) | 62.7(55.6-69.3) |  |
| Smoking status |  |  |  |  |  | <0.001 |
| Never smoker, % | 56.3(50.5-62.0) | 50.9(46.7-55.1) | 53.9(47.4-60.2) | 50.2(45.5-54.9) | 59.6(55.2-63.9) |  |
| Former smoker, % | 17.5(14.2-21.4) | 21.0(17.4-25.1) | 22.4(17.7-27.9) | 28.6(24.2-33.4) | 28.0(24.6-31.8) |  |
| Current smoker, % | 26.2(21.1-32.0) | 28.1(24.2-32.3) | 23.7(19.3-28.8) | 21.2(17.2-25.8) | 12.3(8.9-16.7) |  |
| Drinking status |  |  |  |  |  | 0.001 |
| Nondrinker, % | 35.5(29.1-42.5) | 28.8(22.5-36.0) | 26.9(21.2-33.4) | 23.9(20.3-27.9) | 22.6(18.6-27.2) |  |
| Low-to-moderate drinker, % | 5.6(3.7-8.4) | 11.2(7.7-16.2) | 9.7(7.3-12.8) | 9.1(6.5-12.5) | 14.2(11.7-17.0) |  |
| Heavy drinker, % | 58.9(52.7-64.9) | 60.0(53.7-65.9) | 63.4(56.9-69.4) | 67.1(62.8-71.1) | 63.2(57.9-68.3) |  |
| Physical activity |  |  |  |  |  | 0.019 |
| No activity, % | 37.4(32.9-42.1) | 31.4(28.1-35.0) | 34.6(30.7-38.7) | 28.2(23.7-33.1) | 26.5(22.3-31.3) |  |
| Moderate activity, % | 28.1(24.4-32.2) | 31.9(28.1-35.9) | 33.8(29.1-38.8) | 35.3(29.9-41.0) | 33.5(29.5-37.9) |  |
| Vigorous activity, % | 34.5(29.7-39.5) | 36.7(33.6-39.9) | 31.6(27.3-36.4) | 36.6(31.5-42.0) | 39.9(34.5-45.7) |  |
| Prevalent hypertension, % | 22.1(18.4-26.3) | 30.9(27.3-34.7) | 30.5(26.2-35.1) | 37.1(30.8-43.8) | 32.6(28.2-37.4) | <0.001 |
| Prevalent cardiovascular, % | 6.5(4.3-9.8) | 8.3(6.4-10.7) | 8.8(6.3-12.1) | 10.7(8.3-13.7) | 10.8(7.3-15.8) | 0.171 |
| Prevalent cancer, % | 7.9(5.8-10.7) | 8.7(6.8-11.0) | 7.9(5.2-11.8) | 7.4(5.7-9.5) | 14(9.7-19.8) | 0.016 |

^‡^Values are weighted means (95% CI) or weighted percentages (95% CI).

^*^*P* <0.05. *P* was assessed with General linear models adjusting for age (continuous variables) or χ^2^ test (bivariate relationships).

All data analyses conducted in the current study were based on estimates with sample weights provided by NHANES.

Table S3. Baseline characteristics according to quintiles of serum n-3 Docosapentaenoic acid (DPAn3)^‡^

|  | Serum n-3 Docosapentaenoic acid quintile (%) | | | | | *p*-trend |
| --- | --- | --- | --- | --- | --- | --- |
|  | Q1(<0.32) | Q2(0.32-0.38) | Q3(0.38-0.43) | Q4(0.43-0.49) | Q5(>0.49) |  |
| Age, years | 42.7(41.0-44.5) | 44.7(42.6-46.9) | 45.2(43.4-47.0) | 47.2(45.6-48.8) | 50.3(49.0-51.7) | <0.001 |
| Male, % | 35.2(30.7-40.0) | 52.7(48.1-57.2) | 51.1(45.6-56.6) | 51.1(47.3-54.9) | 49.0(45.0-53.0) | <0.001 |
| Non-Hispanic white | 65.3(58.1-71.9) | 67.5(60.3-73.9) | 70.9(65.4-75.8) | 70.5(63.1-76.9) | 73.5(67.2-79.0) | 0.046 |
| BMI, kg/m^2^ | 29.3(28.6-30.0) | 28.7(28.0-29.3) | 28.5(27.7-29.2) | 27.9(27.2-28.6) | 27.2(26.5-27.9) | <0.001 |
| WAIST, cm | 98.9(96.8-100.9) | 97.9(96.5-99.3) | 98.3(96.4-100.2) | 96.6(94.9-98.3) | 95.8(94.1-97.6) | <0.001 |
| Education |  |  |  |  |  | 0.359 |
| Less than high school, % | 17.1(13.2-21.9) | 19.7(15.7-24.6) | 16.8(14.1-19.7) | 19.8(13.5-28.0) | 15.8(11.9-20.6) |  |
| High school or equivalent, % | 21.6(17.6-26.1) | 23.8(19.5-28.7) | 23.1(19.9-26.7) | 26.3(21.1-32.2) | 21.4(17.3-26.3) |  |
| College graduate or above, % | 61.3(55.6-66.8) | 56.4(50.4-62.3) | 60.1(56.3-63.9) | 53.9(45.9-61.8) | 62.8(57.0-68.3) |  |
| Family income-to-poverty ratio |  |  |  |  |  | 0.233 |
| ≤1.0 | 14.6(12.0-17.7) | 18.0(14.6-22.0) | 15.6(12.4-19.4) | 16.4(12.8-20.8) | 11.5(8.9-14.8) |  |
| 1.0-3.0 | 38.3(32.6-44.4) | 35.6(31.2-40.3) | 36.3(31.3-41.6) | 37.2(31.3-43.6) | 34.2(26.9-42.5) |  |
| >3.0 | 47.0(41.5-52.7) | 46.4(41.2-51.6) | 48.2(42.9-53.4) | 46.4(38.4-54.5) | 54.3(45.7-62.5) |  |
| Smoking status |  |  |  |  |  | 0.008 |
| Never smoker, % | 55.8(50.9-60.7) | 49.2(44.5-54.0) | 53.5(48.5-58.3) | 51.4(45.0-57.8) | 61.0(57.2-64.7) |  |
| Former smoker, % | 22.5(19.4-26.0) | 22.6(18.3-27.4) | 25.8(22.2-29.9) | 23.9(19.5-28.8) | 22.7(19.1-26.8) |  |
| Current smoker, % | 21.7(17.2-27.0) | 28.2(23.6-33.4) | 20.7(16.5-25.6) | 24.7(19.4-31.0) | 16.2(13.1-20.0) |  |
| Drinking status |  |  |  |  |  | 0.117 |
| Nondrinker, % | 28.9(23.6-34.8) | 26.6(21.2-32.8) | 27.1(23.1-31.4) | 28.4(22.6-35.1) | 26.8(21.2-33.3) |  |
| Low-to-moderate drinker, % | 6.5(4.3-9.6) | 8.0(5.9-10.8) | 10.6(7.4-15.0) | 11.9(8.5-16.2) | 13.5(10.4-17.2) |  |
| Heavy drinker, % | 64.6(58.8-70.0) | 65.4(59.9-70.5) | 62.4(57.9-66.6) | 59.8(52.9-66.2) | 59.7(52.7-66.4) |  |
| Physical activity |  |  |  |  |  | 0.648 |
| No activity, % | 34.2(29.8-38.9) | 33.3(29.4-37.4) | 31.3(27.0-35.9) | 29.1(24.4-34.3) | 29.7(24.5-35.5) |  |
| Moderate activity, % | 31.6(27.7-35.8) | 32.3(29.4-35.4) | 33.7(28.9-38.9) | 33.5(28.6-38.9) | 31.6(28.1-35.4) |  |
| Vigorous activity, % | 34.2(28.9-39.8) | 34.4(31.2-37.7) | 35.0(30.2-40.2) | 37.4(32.6-42.4) | 38.7(34.2-43.4) |  |
| Prevalent hypertension, % | 27.2(22.6-32.2) | 31.8(26.6-37.6) | 29.7(25.7-34.1) | 34.1(29.9-38.6) | 31.0(27.1-35.1) | 0.196 |
| Prevalent cardiovascular, % | 6.8(4.2-11.0) | 8.1(6.2-10.4) | 7.8(6.2-9.9) | 11.7(8.9-15.3) | 10.5(7.7-14.1) | 0.058 |
| Prevalent cancer, % | 8.1(5.8-11.1) | 9.8(6.5-14.4) | 7.9(5.1-11.9) | 8.2(5.5-11.9) | 10.9(8.6-13.8) | 0.572 |

^‡^Values are weighted means (95% CI) or weighted percentages (95% CI).

^*^*P* <0.05. *P* was assessed with General linear models adjusting for age (continuous variables) or χ^2^ test (bivariate relationships).

All data analyses conducted in the current study were based on estimates with sample weights provided by NHANES.

Table S4. Baseline characteristics according to quintiles of serum docosahexaenoic acid (DHA)^‡^

|  | Serum docosahexaenoic acid quintile (%) | | | | | *p*-trend |
| --- | --- | --- | --- | --- | --- | --- |
|  | Q1(<0.87) | Q2(0.87-1.09) | Q3(1.09-1.33) | Q4(1.33-1.72) | Q5(>1.72) |  |
| Age, years | 42.6(41.6-43.7) | 44.3(42.4-46.2) | 46.3(44.6-48.0) | 47.5(45.4-49.7) | 51.5(49.5-53.5) | <0.001 |
| Male, % | 58.8(54.8-62.8) | 51.0(46.3-55.8) | 44.3(39.2-49.5) | 41.4(37.2-45.8) | 38.6(34.1-43.3) | <0.001 |
| Non-Hispanic white | 78.5(72.0-83.9) | 70.6(63.8-76.6) | 70.0(63.6-75.7) | 62.4(55.3-68.9) | 61.8(54.4-68.7) | <0.001 |
| BMI, kg/m^2^ | 28.9(28.3-29.6) | 28.9(28.2-29.5) | 28.5(27.9-29.2) | 28.3(27.3-29.3) | 26.3(25.7-26.9) | <0.001 |
| WAIST, cm | 99.9(98.1-101.7) | 98.6(97.2-100.0) | 97.9(96.3-99.5) | 96.7(94.3-99.0) | 92.7(91.0-94.3) | <0.001 |
| Education |  |  |  |  |  | <0.001 |
| Less than high school, % | 20.3(16.2-25.0) | 22.5(17.3-28.6) | 16.1(12.5-20.5) | 16.4(12.9-20.6) | 12.0(8.9-16.0) |  |
| High school or equivalent, % | 25.8(22.3-29.7) | 26.4(22.3-31.1) | 26.2(21.9-31.1) | 18.9(14.9-23.8) | 16.4(12.4-21.5) |  |
| College graduate or above, % | 53.9(49.0-58.7) | 51.1(45.0-57.1) | 57.7(51.8-63.3) | 64.7(59.3-69.7) | 71.5(65.9-76.6) |  |
| Family income-to-poverty ratio |  |  |  |  |  | <0.001 |
| ≤1.0 | 18.3(15.0-22.0) | 15.9(11.7-21.1) | 17.2(14.0-21.1) | 13.7(10.9-17.0) | 9.5(7.1-12.6) |  |
| 1.0-3.0 | 40.3(35.5-45.3) | 41.0(35.5-46.6) | 33.7(29.4-38.2) | 35.5(30.4-41.1) | 28.1(21.8-35.2) |  |
| >3.0 | 41.4(36.9-46.1) | 43.2(36.9-49.7) | 49.1(43.7-54.4) | 50.8(44.4-57.2) | 62.4(54.2-70.0) |  |
| Smoking status |  |  |  |  |  | <0.001 |
| Never smoker, % | 45.3(40.0-50.7) | 49.3(43.9-54.7) | 57.4(53.2-61.6) | 58.9(54.3-63.3) | 64.1(59.6-68.3) |  |
| Former smoker, % | 18.0(14.9-21.6) | 23.4(19.3-28.1) | 24.9(21.4-28.8) | 25.7(21.2-30.7) | 28.2(24.0-32.8) |  |
| Current smoker, % | 36.7(31.6-42.2) | 27.3(22.7-32.3) | 17.7(13.9-22.3) | 15.5(11.8-20.0) | 7.8(5.8-10.2) |  |
| Drinking status |  |  |  |  |  | 0.389 |
| Nondrinker, % | 28.0(21.5-35.5) | 30.3(25.0-36.1) | 25.2(20.5-30.5) | 29.0(23.9-34.8) | 24.6(21.0-28.6) |  |
| Low-to-moderate drinker, % | 9.3(7.3-11.8) | 9.9(6.6-14.6) | 9.3(5.7-14.9) | 8.3(5.6-12.1) | 13.8(10.7-17.5) |  |
| Heavy drinker, % | 62.7(55.6-69.4) | 59.9(54.2-65.2) | 65.5(59.5-71.1) | 62.7(56.8-68.3) | 61.6(57.1-66.0) |  |
| Physical activity |  |  |  |  |  | 0.285 |
| No activity, % | 31.3(26.5-36.5) | 34.4(29.0-40.3) | 31.2(27.8-34.9) | 33.0(28.7-37.6) | 27.0(22.6-32.0) |  |
| Moderate activity, % | 34.4(30.8-38.2) | 30.4(26.9-34.1) | 34.7(29.6-40.1) | 31.7(27.9-35.8) | 31.1(27.2-35.3) |  |
| Vigorous activity, % | 34.3(29.4-39.5) | 35.2(30.4-40.3) | 34.1(29.7-38.9) | 35.3(29.4-41.7) | 41.9(36.0-47.9) |  |
| Prevalent hypertension, % | 28.8(24.4-33.6) | 30.1(25.8-34.8) | 30.2(25.5-35.4) | 34.9(30.2-40.0) | 30.9(26.3-35.8) | 0.330 |
| Prevalent cardiovascular, % | 7.1(5.3-9.4) | 8.1(5.9-11.0) | 9.4(7.0-12.6) | 12.0(8.8-16.1) | 9.6(6.3-14.3) | 0.118 |
| Prevalent cancer, % | 7.1(4.7-10.5) | 8.1(6.0-10.7) | 9.2(6.2-13.4) | 9.9(7.1-13.6) | 12.6(8.9-17.6) | 0.174 |

^‡^Values are weighted means (95% CI) or weighted percentages (95% CI).

^*^*P* <0.05. *P* was assessed with General linear models adjusting for age (continuous variables) or χ^2^ test (bivariate relationships).

All data analyses conducted in the current study were based on estimates with sample weights provided by NHANES.

Table S5. Baseline characteristics according to quintiles of serum myristic acid (MA)^‡^

|  | Serum myristic acid quintile (%) | | | | | *p*-trend |
| --- | --- | --- | --- | --- | --- | --- |
|  | Q1(<0.03) | Q2(0.03-0.04) | Q3(0.04-0.06) | Q4(0.06-0.09) | Q5(>0.09) |  |
| Age, years | 45.7(43.7-47.7) | 47.1(45.0-49.2) | 44.9(43.2-46.6) | 47.6(46.2-48.9) | 45.3(43.8-46.9) | 0.881 |
| Male, % | 55.6(51.1-60.1) | 46.7(42.0-51.6) | 44.2(40.1-48.4) | 46.1(40.9-51.4) | 47.5(42.5-52.5) | 0.032 |
| Non-Hispanic white | 49.9(41.5-58.4) | 67.9(59.4-75.3) | 73.1(67.8-77.8) | 74.0(67.8-79.4) | 79.2(73.8-83.8) | <0.001 |
| BMI, kg/m^2^ | 27.2(26.6-27.8) | 27.1(26.4-27.9) | 28.1(27.4-28.9) | 29.2(28.6-29.8) | 29.5(29.1-30.0) | <0.001 |
| WAIST, cm | 94.2(92.7-95.8) | 94.6(92.7-96.5) | 96.5(94.6-98.4) | 99.9(98.2-101.7) | 101.3(99.9-102.8) | <0.001 |
| Education |  |  |  |  |  | 0.383 |
| Less than high school, % | 21.4(16.8-26.8) | 20.7(15.9-26.4) | 16.6(12.4-21.9) | 18.2(15.3-21.5) | 15.6(12.5-19.2) |  |
| High school or equivalent, % | 21.0(16.6-26.3) | 23.2(19.0-28.1) | 24.5(20.4-29.2) | 23.6(20.2-27.3) | 24.1(19.9-28.9) |  |
| College graduate or above, % | 57.6(53.0-62.0) | 56.1(48.9-63.0) | 58.8(52.3-65.0) | 58.3(53.1-63.2) | 60.3(54.2-66.2) |  |
| Family income-to-poverty ratio |  |  |  |  |  | 0.595 |
| ≤1.0 | 17.1(13.3-21.8) | 17.0(13.6-21.0) | 15.0(11.6-19.2) | 14.0(10.8-17.9) | 13.9(10.4-18.3) |  |
| 1.0-3.0 | 36.1(30.7-41.9) | 33.5(28.7-38.6) | 35.1(30.0-40.5) | 38.6(33.7-43.8) | 37.3(32.9-41.9) |  |
| >3.0 | 46.8(40.7-52.9) | 49.5(43.7-55.4) | 49.9(44.4-55.4) | 47.4(42.0-52.9) | 48.8(43.0-54.7) |  |
| Smoking status |  |  |  |  |  | <0.001 |
| Never smoker, % | 60.0(55.3-64.5) | 54.3(48.3-60.1) | 58.2(53.1-63.1) | 52.1(47.0-57.1) | 46.2(40.9-51.6) |  |
| Former smoker, % | 23.5(18.7-29.1) | 26.5(22.2-31.4) | 18.1(14.4-22.6) | 24.9(20.7-29.7) | 25.6(20.2-31.9) |  |
| Current smoker, % | 16.5(13.1-20.5) | 19.2(15.1-24.0) | 23.7(19.6-28.4) | 23.0(19.0-27.5) | 28.2(23.9-32.8) |  |
| Drinking status |  |  |  |  |  | 0.003 |
| Nondrinker, % | 31.3(25.6-37.5) | 30.8(25.0-37.3) | 26.1(20.8-32.1) | 26.8(20.8-33.7) | 25.0(20.2-30.5) |  |
| Low-to-moderate drinker, % | 14.1(10.7-18.3) | 12.3(9.6-15.7) | 10.0(7.6-13.0) | 6.8(4.7-9.7) | 7.6(5.5-10.4) |  |
| Heavy drinker, % | 54.7(49.2-60.0) | 56.9(50.7-62.8) | 64.0(58.2-69.4) | 66.5(59.1-73.2) | 67.4(62.5-72.0) |  |
| Physical activity |  |  |  |  |  | 0.849 |
| No activity, % | 32.6(28.4-37.2) | 32.1(27.9-36.5) | 33.2(29.7-37.0) | 31.8(26.3-37.9) | 30.9(26.3-35.9) |  |
| Moderate activity, % | 29.7(24.9-35.1) | 30.0(25.7-34.8) | 31.4(27.3-35.9) | 33.6(28.2-39.4) | 34.1(30.2-38.2) |  |
| Vigorous activity, % | 37.7(33.3-42.2) | 37.9(32.8-43.4) | 35.4(30.3-40.8) | 34.6(29.3-40.3) | 35.0(30.8-39.5) |  |
| Prevalent hypertension, % | 28.0(24.7-31.6) | 27.9(23.5-32.8) | 28.2(23.4-33.4) | 33.6(28.6-39.0) | 33.4(28.5-38.7) | 0.111 |
| Prevalent cardiovascular, % | 9.5(7.1-12.7) | 8.1(5.2-12.4) | 7.8(5.6-10.9) | 10.9(7.7-15.3) | 8.9(6.8-11.7) | 0.481 |
| Prevalent cancer, % | 8.7(6.2-12.1) | 10.6(8-14) | 8.1(6.3-10.4) | 9.6(7.7-12) | 8.7(5.9-12.7) | 0.681 |

^‡^Values are weighted means (95% CI) or weighted percentages (95% CI).

^*^*P* <0.05. *P* was assessed with General linear models adjusting for age (continuous variables) or χ^2^ test (bivariate relationships).

All data analyses conducted in the current study were based on estimates with sample weights provided by NHANES.

Table S6. Baseline characteristics according to quintiles of serum linoleic acid (LA)^‡^

|  | Serum linoleic acid quintile (%) | | | | | *p*-trend |
| --- | --- | --- | --- | --- | --- | --- |
|  | Q1(<27.71) | Q2(27.71-30.7) | Q3(30.7-32.96) | Q4(32.96-35.63) | Q5(>35.63) |  |
| Age, years | 51.6(49.8-53.3) | 48.7(47.2-50.2) | 46.2(44.5-47.9) | 44.2(42.1-46.3) | 39.7(37.1-42.4) | <0.001 |
| Male, % | 54.3(49.1-59.5) | 51.0(47.2-54.7) | 47.4(42.4-52.5) | 42.1(37.2-47.2) | 45.0(40.7-49.3) | 0.007 |
| Non-Hispanic white | 75.0(69.5-79.8) | 74.1(68.6-79.0) | 69.5(62.5-75.7) | 69.4(62.7-75.4) | 59.9(52.1-67.3) | <0.001 |
| BMI, kg/m^2^ | 30.1(29.3-30.9) | 29.0(28.4-29.6) | 28.4(27.7-29.1) | 27.5(26.8-28.1) | 26.6(26.0-27.1) | <0.001 |
| WAIST, cm | 104(101.9-106.1) | 100.2(98.5-101.9) | 97.5(95.7-99.2) | 94.6(93.1-96.2) | 91.7(90.3-93.0) | <0.001 |
| Education |  |  |  |  |  | 0.276 |
| Less than high school, % | 18.5(15.8-21.4) | 16.8(12.8-21.9) | 19.2(14.9-24.4) | 17.1(13.9-20.9) | 17.8(13.5-23.0) |  |
| High school or equivalent, % | 27.0(22.7-31.9) | 23.4(18.8-28.6) | 23.6(19.9-27.7) | 19.6(15.1-24.9) | 23.3(19.2-28.0) |  |
| College graduate or above, % | 54.5(49.3-59.6) | 59.8(53.1-66.1) | 57.2(52.8-61.5) | 63.4(57.2-69.1) | 59.0(52.0-65.6) |  |
| Family income-to-poverty ratio |  |  |  |  |  | 0.212 |
| ≤1.0 | 13.5(10.8-16.7) | 13.0(10.0-16.7) | 14.8(11.4-19.0) | 17.1(13.7-21.2) | 18.3(14.3-23.2) |  |
| 1.0-3.0 | 38.5(32.9-44.4) | 37.1(31.7-42.8) | 38.3(33.1-43.7) | 31.8(27.8-36.2) | 35.7(29.6-42.3) |  |
| >3.0 | 48.0(41.6-54.4) | 49.9(43.7-56.2) | 46.9(40.1-53.9) | 51.0(44.7-57.3) | 46.0(39.4-52.7) |  |
| Smoking status |  |  |  |  |  | <0.001 |
| Never smoker, % | 40.8(36.0-45.8) | 50.2(45.0-55.3) | 56.1(50.9-61.2) | 55.9(50.2-61.4) | 67.2(62.1-71.9) |  |
| Former smoker, % | 29.3(25.5-33.5) | 25.9(22.0-30.3) | 19.8(16.0-24.2) | 22.6(18.9-26.7) | 20.4(16.9-24.4) |  |
| Current smoker, % | 29.9(26.3-33.7) | 23.9(19.4-29.1) | 24.1(19.6-29.3) | 21.5(16.8-27.2) | 12.4(9.6-15.9) |  |
| Drinking status |  |  |  |  |  | 0.178 |
| Nondrinker, % | 27.2(22.9-32.0) | 26.0(20.3-32.7) | 27.5(22.9-32.7) | 25.9(21.7-30.5) | 31.1(25.6-37.2) |  |
| Low-to-moderate drinker, % | 7.8(5.4-11.2) | 9.1(6.8-12.0) | 11.8(8.8-15.7) | 9.6(7.3-12.5) | 11.3(8.6-14.7) |  |
| Heavy drinker, % | 65.0(60.5-69.3) | 64.9(58.7-70.6) | 60.7(56.1-65.1) | 64.6(59.7-69.1) | 57.6(51.4-63.6) |  |
| Physical activity |  |  |  |  |  | 0.003 |
| No activity, % | 36.6(30.8-42.9) | 30.1(25.5-35.2) | 34.5(30.8-38.3) | 31.4(28.6-34.3) | 25.6(22.1-29.4) |  |
| Moderate activity, % | 35.7(30.7-41.0) | 35.0(30.3-40.1) | 29.8(24.7-35.4) | 28.7(24.4-33.5) | 33.8(28.8-39.1) |  |
| Vigorous activity, % | 27.7(24.5-31.1) | 34.8(30.2-39.8) | 35.8(30.3-41.6) | 39.9(35.4-44.5) | 40.7(36.2-45.3) |  |
| Prevalent hypertension, % | 45.5(40.2-51.0) | 35.2(29.8-41.0) | 28.5(23.5-34.2) | 26.8(21.8-32.4) | 18.6(15.4-22.3) | <0.001 |
| Prevalent cardiovascular, % | 17.9(14.7-21.6) | 10.3(7.4-14.1) | 10.4(7.7-14) | 4.1(2.2-7.4) | 2.9(1.8-4.7) | <0.001 |
| Prevalent cancer, % | 11.5(9.0-14.6) | 9.2(6.8-12.3) | 9(6.3-12.7) | 9.9(6.9-14.1) | 5.9(3.7-9.4) | 0.141 |

^‡^Values are weighted means (95% CI) or weighted percentages (95% CI).

^*^*P* <0.05. *P* was assessed with General linear models adjusting for age (continuous variables) or χ^2^ test (bivariate relationships).

All data analyses conducted in the current study were based on estimates with sample weights provided by NHANES.

Table S7. Baseline characteristics according to quintiles of serum gamma-linoleic acid (GLA)^‡^

|  | Serum gamma-linoleic acid quintile (%) | | | | | *p*-trend |
| --- | --- | --- | --- | --- | --- | --- |
|  | Q1(<0.3) | Q2(0.3-0.39) | Q3(0.39-0.48) | Q4(0.48-0.61) | Q5(>0.61) |  |
| Age, years | 42.8(41.0-44.6) | 44.6(42.6-46.7) | 46.0(44.1-48) | 48.1(46.4-49.9) | 47.7(46.3-49) | <0.001 |
| Male, % | 41.1(36.7-45.7) | 50.7(46.5-54.9) | 48.9(42.9-54.9) | 50.5(46.3-54.7) | 48.2(43.6-52.9) | 0.065 |
| Non-Hispanic white | 57.5(49.0-65.5) | 66.0(59.4-72.1) | 69.6(63.3-75.3) | 73.5(67.4-78.9) | 77.5(70.8-83.0) | <0.001 |
| BMI, kg/m^2^ | 26.6(26.0-27.2) | 28.2(27.4-29.0) | 28.6(28.0-29.3) | 28.6(27.9-29.3) | 29.0(28.5-29.5) | <0.001 |
| WAIST, cm | 92.8(91.0-94.6) | 97.1(94.9-99.3) | 98.0(96.3-99.7) | 98.9(97.4-100.4) | 99.6(98.6-100.6) | <0.001 |
| Education |  |  |  |  |  | 0.070 |
| Less than high school, % | 18.0(14.3-22.5) | 21.5(17.2-26.6) | 17.0(12.3-22.9) | 15.9(12.8-19.6) | 18.0(14.4-22.3) |  |
| High school or equivalent, % | 18.3(14.7-22.5) | 22.4(18.6-26.6) | 22.9(18.0-28.7) | 23.4(18.7-29.0) | 27.1(22.8-31.8) |  |
| College graduate or above, % | 63.7(58.8-68.3) | 56.1(50.6-61.4) | 60.1(53.2-66.6) | 60.6(54.3-66.6) | 54.9(49.4-60.4) |  |
| Family income-to-poverty ratio |  |  |  |  |  | 0.198 |
| ≤1.0 | 16.7(13.3-20.7) | 16.6(12.8-21.4) | 14.3(10.5-19.2) | 13.3(10.0-17.5) | 16.2(13.4-19.4) |  |
| 1.0-3.0 | 36.8(31.6-42.3) | 37.4(31.5-43.8) | 33.5(28.9-38.5) | 33.6(30.2-37.3) | 39.1(32.8-45.8) |  |
| >3.0 | 46.5(41.0-52.1) | 45.9(39.7-52.3) | 52.2(46.3-58.1) | 53.1(48.3-57.8) | 44.7(37.6-52.0) |  |
| Smoking status |  |  |  |  |  | 0.002 |
| Never smoker, % | 63.7(58.6-68.4) | 52.8(46.4-59.2) | 54.0(50.2-57.9) | 51.4(46.7-56.0) | 50.5(45.1-55.9) |  |
| Former smoker, % | 22.4(18.4-27.0) | 26.0(20.9-31.7) | 24.6(20.7-29.1) | 23.2(19.2-27.6) | 22.9(18.9-27.3) |  |
| Current smoker, % | 13.9(10.6-18.1) | 21.2(18.5-24.2) | 21.3(16.8-26.6) | 25.4(20.4-31.3) | 26.6(21.8-32.1) |  |
| Drinking status |  |  |  |  |  | 0.594 |
| Nondrinker, % | 25.9(21.3-31) | 32.1(24.5-40.7) | 26.2(21.3-31.9) | 25.9(21.2-31.3) | 26.5(21.5-32.2) |  |
| Low-to-moderate drinker, % | 9.6(6.7-13.6) | 10.1(7.7-13.2) | 10.9(8.4-14.1) | 10.5(7.6-14.2) | 9.2(6.6-12.7) |  |
| Heavy drinker, % | 64.5(59.7-69) | 57.8(49.5-65.7) | 62.8(58.1-67.4) | 63.6(56.9-69.8) | 64.3(58.6-69.6) |  |
| Physical activity |  |  |  |  |  | 0.542 |
| No activity, % | 34.7(30.2-39.4) | 31.7(27.4-36.3) | 31.0(25.8-36.7) | 33.6(29.5-38.0) | 28.0(23.4-33.1) |  |
| Moderate activity, % | 28.4(23.5-33.9) | 33.5(29.5-37.8) | 33.7(29.8-37.8) | 31.7(26.6-37.4) | 33.8(29.9-37.9) |  |
| Vigorous activity, % | 36.9(30.5-43.8) | 34.8(29.4-40.5) | 35.3(30.6-40.4) | 34.6(29.7-39.9) | 38.2(33.5-43.2) |  |
| Prevalent hypertension, % | 22.9(19.0-27.5) | 29.0(24.3-34.1) | 31.1(26.8-35.8) | 32.6(28.4-37.2) | 35.3(30.3-40.6) | 0.002 |
| Prevalent cardiovascular, % | 4.4(3.3-5.9) | 9.2(7-11.9) | 9.2(6.7-12.6) | 9.8(7.6-12.6) | 11.9(8.8-16.1) | <0.001 |
| Prevalent cancer, % | 8.1(5.9-11.0) | 8.9(6.2-12.6) | 9.2(6.6-12.7) | 8.8(6.7-11.5) | 10.8(7.1-15.9) | 0.736 |

^‡^Values are weighted means (95% CI) or weighted percentages (95% CI).

^*^*P* <0.05. *P* was assessed with General linear models adjusting for age (continuous variables) or χ^2^ test (bivariate relationships).

All data analyses conducted in the current study were based on estimates with sample weights provided by NHANES.

Table S8. Baseline characteristics according to quintiles of serum dihomo-gamma-linoleic acid (DGLA)^‡^

|  | Serum dihomo-gamma-linoleic acid quintile (%) | | | | | *p*-trend |
| --- | --- | --- | --- | --- | --- | --- |
|  | Q1(<1.07) | Q2(1.07-1.24) | Q3(1.24-1.4) | Q4(1.4-1.62) | Q5(>1.62) |  |
| Age, years | 47.3(45.4-49.2) | 48.0(46.3-49.7) | 45.0(43.4-46.5) | 45.2(43.6-46.9) | 44.2(42.3-46.0) | 0.001 |
| Male, % | 55.0(49.8-60.0) | 54.3(49.1-59.3) | 49.3(44.7-54.0) | 44.1(39.5-48.8) | 39.1(34.1-44.3) | <0.001 |
| Non-Hispanic white | 64.5(56.7-71.5) | 70.8(63.9-76.8) | 66.6(59.6-73.1) | 71.8(65.8-77.1) | 72.1(66.4-77.3) | 0.026 |
| BMI, kg/m^2^ | 27.0(26.4-27.7) | 27.6(27-28.1) | 28.1(27.5-28.7) | 28.7(28.1-29.4) | 29.6(28.9-30.3) | <0.001 |
| WAIST, cm | 95.4(93.7-97.0) | 96.6(95.2-98) | 97.1(95.4-98.9) | 97.8(96.1-99.5) | 99.5(97.9-101.2) | <0.001 |
| Education |  |  |  |  |  | 0.676 |
| Less than high school, % | 18.0(13.7-23.3) | 15.2(11.8-19.4) | 17.6(13.9-22.1) | 17.5(13.8-21.9) | 19.5(15.9-23.6) |  |
| High school or equivalent, % | 21.7(17.4-26.6) | 24.5(19.4-30.5) | 22.3(18.3-27.1) | 25.4(21.1-30.1) | 21.9(17.2-27.5) |  |
| College graduate or above, % | 60.3(53.3-67.0) | 60.2(53.4-66.7) | 60.0(54.2-65.6) | 57.1(52.1-62.0) | 58.6(52.2-64.8) |  |
| Family income-to-poverty ratio |  |  |  |  |  | 0.296 |
| ≤1.0 | 14.4(11.5-17.9) | 16.6(12.6-21.6) | 14.2(10.5-19.0) | 14.4(12.2-17.0) | 15.6(11.7-20.4) |  |
| 1.0-3.0 | 39.3(32.1-46.9) | 30.8(25.4-36.8) | 38.3(32.7-44.2) | 33.6(29.1-38.5) | 37.7(32.1-43.7) |  |
| >3.0 | 46.3(39.1-53.7) | 52.6(46.0-59.1) | 47.5(41.1-53.9) | 51.9(46.9-56.9) | 46.7(40.5-53.0) |  |
| Smoking status |  |  |  |  |  | 0.573 |
| Never smoker, % | 56.8(50.6-62.8) | 51.6(46.9-56.2) | 55.1(49.5-60.4) | 52.1(48.0-56.2) | 56.7(53.4-59.9) |  |
| Former smoker, % | 22.8(19.2-26.8) | 25.4(22.0-29.2) | 23.8(20.7-27.3) | 23.9(19.5-29.0) | 21.3(17.9-25.0) |  |
| Current smoker, % | 20.4(16.2-25.4) | 23.0(18.3-28.5) | 21.1(16.6-26.5) | 24.0(20.4-28.1) | 22.1(17.8-27.0) |  |
| Drinking status |  |  |  |  |  | 0.016 |
| Nondrinker, % | 27.2(21.4-33.9) | 26.1(21.4-31.5) | 29.0(24.7-33.7) | 25.7(20.2-32.0) | 29.6(24.7-34.9) |  |
| Low-to-moderate drinker, % | 12.8(10.0-16.3) | 11.4(8.7-14.8) | 12.9(9.9-16.7) | 8.2(6.0-11.1) | 6.2(3.9-9.8) |  |
| Heavy drinker, % | 60.0(54.3-65.4) | 62.4(56.7-67.8) | 58.1(54.2-61.9) | 66.1(60.0-71.7) | 64.2(58.7-69.3) |  |
| Physical activity |  |  |  |  |  | 0.931 |
| No activity, % | 30.9(26.6-35.5) | 30.7(26.0-35.9) | 31.9(28.1-35.9) | 30.8(27.2-34.8) | 31.6(27.5-36.0) |  |
| Moderate activity, % | 35.1(30.6-39.9) | 33.0(28.5-37.7) | 30.6(25.8-35.8) | 32.9(29.0-37.1) | 31.7(27.6-36.0) |  |
| Vigorous activity, % | 34.0(29.4-38.9) | 36.3(30.7-42.3) | 37.5(32.2-43.2) | 36.3(32.4-40.3) | 36.7(31.9-41.9) |  |
| Prevalent hypertension, % | 33.4(28.2-39.1) | 33.6(29.4-38.0) | 27.2(22.6-32.5) | 28.5(24.9-32.3) | 29.9(25.2-35.1) | 0.130 |
| Prevalent cardiovascular, % | 11.3(8.2-15.3) | 10.3(7.9-13.3) | 8.0(6.1-10.5) | 9.1(6.3-13.1) | 6.1(4.2-8.7) | 0.050 |
| Prevalent cancer, % | 10.5(8.3-13.4) | 8.8(6.2-12.2) | 8.4(6-11.7) | 8.7(6.1-12.3) | 8.1(6.3-10.3) | 0.686 |

^‡^Values are weighted means (95% CI) or weighted percentages (95% CI).

^*^*P* <0.05. *P* was assessed with General linear models adjusting for age (continuous variables) or χ^2^ test (bivariate relationships).

All data analyses conducted in the current study were based on estimates with sample weights provided by NHANES.

Table S9. Baseline characteristics according to quintiles of serum arachidonic acid (AA)^‡^

|  | Serum arachidonic acid quintile (%) | | | | | *p*-trend |
| --- | --- | --- | --- | --- | --- | --- |
|  | Q1(<5.74) | Q2(5.74-6.77) | Q3(6.77-7.72) | Q4(7.72-8.91) | Q5(>8.91) |  |
| Age, years | 46.5(45.0-47.9) | 44.9(43.0-46.9) | 45.2(43.6-46.8) | 45.2(43.6-46.8) | 49.1(47.3-50.9) | 0.090 |
| Male, % | 54.5(49.8-59.1) | 46.7(42.4-51.1) | 47.4(42.1-52.6) | 45.9(41.9-49.9) | 44.9(40-.049.9) | 0.060 |
| Non-Hispanic white | 65.3(57.8-72.2) | 74.0(68.2-79.1) | 74.4(68.5-79.4) | 72.2(65.5-78.0) | 58.2(49.3-66.6) | <0.001 |
| BMI, kg/m^2^ | 28.8(28.1-29.5) | 28.3(27.9-28.8) | 27.5(26.8-28.1) | 28.0(27.5-28.6) | 29.0(28.4-29.7) | 0.835 |
| WAIST, cm | 100.1(98.1-102.2) | 97.9(96.6-99.3) | 95.2(93.4-97.0) | 96.4(95.2-97.6) | 98.4(96.7-100.0) | 0.023 |
| Education |  |  |  |  |  | 0.497 |
| Less than high school, % | 19.3(15.6-23.7) | 17.7(14.2-22.0) | 17.3(12.9-22.9) | 16.3(12.8-20.5) | 19.2(14.4-25.1) |  |
| High school or equivalent, % | 19.1(15.7-23.0) | 23.9(19.6-28.7) | 25.0(19.9-30.9) | 23.9(19.8-28.5) | 24.5(21.2-28.1) |  |
| College graduate or above, % | 61.6(55.8-67.1) | 58.4(52.8-63.8) | 57.7(50.9-64.2) | 59.9(54.7-64.9) | 56.4(51.1-61.5) |  |
| Family income-to-poverty ratio |  |  |  |  |  | 0.901 |
| ≤1.0 | 14.1(10.9-17.9) | 14.2(11.9-16.9) | 15.9(11.7-21.4) | 15.7(12.2-19.9) | 17.3(13.7-21.6) |  |
| 1.0-3.0 | 36.4(31.2-42.0) | 36.6(31.8-41.8) | 35.1(30.5-40.0) | 36.8(31.4-42.5) | 36.6(30.5-43.1) |  |
| >3.0 | 49.5(44.5-54.6) | 49.2(43.9-54.4) | 49.0(41.7-56.3) | 47.6(41.2-54.0) | 46.1(39.4-53.0) |  |
| Smoking status |  |  |  |  |  | 0.515 |
| Never smoker, % | 50.7(45.5-55.9) | 53.7(47.9-59.4) | 52.6(46.5-58.6) | 54.8(50.9-58.6) | 59.8(54.0-65.3) |  |
| Former smoker, % | 25.1(20.6-30.1) | 24.5(20.7-28.7) | 23.2(18.7-28.4) | 23.8(20.1-28.1) | 20.8(16.5-25.9) |  |
| Current smoker, % | 24.2(19.7-29.4) | 21.8(17.9-26.2) | 24.2(18.7-30.8) | 21.4(17.4-26.0) | 19.4(15.3-24.3) |  |
| Drinking status |  |  |  |  |  | 0.224 |
| Nondrinker, % | 30.6(24.2-37.7) | 28.8(22.5-36.0) | 26.0(21.3-31.3) | 23.4(19.1-28.4) | 29.8(24.3-35.9) |  |
| Low-to-moderate drinker, % | 11.2(8.6-14.5) | 8.0(5.6-11.4) | 8.7(6.1-12.1) | 12.2(8.9-16.4) | 10.0(7.0-14.0) |  |
| Heavy drinker, % | 58.2(51.0-65.1) | 63.2(56.0-69.9) | 65.4(60.3-70.1) | 64.4(60.0-68.6) | 60.3(54.1-66.1) |  |
| Physical activity |  |  |  |  |  | 0.493 |
| No activity, % | 32.6(27.9-37.7) | 30.5(26.3-35.2) | 30.5(25.9-35.5) | 31.6(27.8-35.7) | 32.9(28.4-37.9) |  |
| Moderate activity, % | 34.4(30.1-38.9) | 32.8(29.2-36.8) | 29.5(26.3-33.0) | 33.0(28.9-37.4) | 33.8(29.4-38.4) |  |
| Vigorous activity, % | 33.0(28.7-37.7) | 36.6(31.5-42.0) | 40.0(35.4-44.7) | 35.4(31.0-40.1) | 33.3(27.9-39.1) |  |
| Prevalent hypertension, % | 34.5(28.7-40.8) | 25.7(21.3-30.6) | 29.2(24.3-34.7) | 27.9(23.7-32.5) | 39.2(34.8-43.8) | 0.001 |
| Prevalent cardiovascular, % | 9.4(6.7-13.0) | 7.1(5.0-10.0) | 7.1(5.2-9.6) | 9.9(7.6-12.8) | 12.8(9.4-17.1) | 0.023 |
| Prevalent cancer, % | 9.7(6.9-13.5) | 10.8(7.7-15.1) | 6.6(4.7-9.2) | 9.4(6.2-14.0) | 9.3(6.5-13.1) | 0.395 |

^‡^Values are weighted means (95% CI) or weighted percentages (95% CI).

^*^*P* <0.05. *P* was assessed with General linear models adjusting for age (continuous variables) or χ^2^ test (bivariate relationships).

All data analyses conducted in the current study were based on estimates with sample weights provided by NHANES.

Table S10. Baseline characteristics according to quintiles of serum eicosadienoic acid (EDA)^‡^

|  | Serum eicosadienoic acid quintile (%) | | | | | *p*-trend |
| --- | --- | --- | --- | --- | --- | --- |
|  | Q1(<0.16) | Q2(0.16-0.18) | Q3(0.18-0.2) | Q4(0.2-0.23) | Q5(>0.23) |  |
| Age, years | 46.0(44.5-47.5) | 45.7(43.9-47.5) | 46.8(45.0-48.6) | 46.7(44.7-48.6) | 44.0(42.0-46.1) | 0.355 |
| Male, % | 53.5(47.6-59.3) | 47.8(44.1-51.6) | 49.7(45.7-53.7) | 48.9(44.4-53.5) | 39.6(35.0-44.4) | 0.002 |
| Non-Hispanic white | 80.5(75.5-84.7) | 73.9(67.6-79.4) | 70.0(63.5-75.7) | 59.0(50.5-67.0) | 58.9(51.4-66.1) | <0.001 |
| BMI, kg/m^2^ | 28.3(27.8-28.9) | 28.5(27.9-29.2) | 28.8(28.2-29.4) | 28.5(27.9-29.1) | 27.1(26.6-27.7) | 0.008 |
| WAIST, cm | 98.5(97.1-100.0) | 97.6(96.2-99.0) | 98.8(97.2-100.3) | 97.7(96.4-99.1) | 93.8(92.1-95.5) | <0.001 |
| Education |  |  |  |  |  | <0.001 |
| Less than high school, % | 14.0(10.9-17.7) | 12.4(9.9-15.4) | 20.6(16.3-25.7) | 20.4(15.8-25.8) | 24.6(20.2-29.6) |  |
| High school or equivalent, % | 24.8(20.0-30.3) | 24.4(18.9-31.0) | 23.9(19.2-29.2) | 23.4(20.0-27.2) | 17.9(14.6-21.8) |  |
| College graduate or above, % | 61.2(54.7-67.4) | 63.1(55.8-69.9) | 55.5(50.7-60.2) | 56.2(51.1-61.2) | 57.5(51.6-63.2) |  |
| Family income-to-poverty ratio |  |  |  |  |  | 0.056 |
| ≤1.0 | 13.6(10.2-17.8) | 13.7(10.2-18.1) | 14.3(11.3-17.9) | 16.0(11.8-21.3) | 18.4(14.9-22.6) |  |
| 1.0-3.0 | 33.4(27.4-40.0) | 34.2(27.9-41.1) | 35.4(29.8-41.5) | 40.2(34.4-46.4) | 39.7(35.6-43.8) |  |
| >3.0 | 53.0(46.4-59.5) | 52.1(45.4-58.8) | 50.3(43.8-56.7) | 43.8(37.3-50.6) | 41.9(37.8-46.1) |  |
| Smoking status |  |  |  |  |  | 0.001 |
| Never smoker, % | 46.5(41.1-51.9) | 59.3(53.5-65.0) | 49.9(45.2-54.6) | 52.2(45.5-58.8) | 62.8(58.3-67.1) |  |
| Former smoker, % | 24.9(20.5-29.8) | 21.0(16.3-26.6) | 26.3(21.1-32.2) | 24.9(20.6-29.8) | 20.0(16.4-24.1) |  |
| Current smoker, % | 28.7(24.1-33.8) | 19.6(15.5-24.5) | 23.8(19.9-28.2) | 22.9(18.5-28.0) | 17.2(13.2-22.2) |  |
| Drinking status |  |  |  |  |  | 0.436 |
| Nondrinker, % | 24.4(20.1-29.3) | 28.0(23.2-33.4) | 27.5(22.5-33) | 28.6(22.6-35.5) | 31.5(24.5-39.4) |  |
| Low-to-moderate drinker, % | 9.0(6.7-12.1) | 9.9(7.8-12.7) | 11.7(8.5-16.0) | 11.4(8.0-16.2) | 8.6(5.4-13.4) |  |
| Heavy drinker, % | 66.5(62.0-70.8) | 62.0(56.5-67.3) | 60.8(55.6-65.7) | 60.0(53.1-66.5) | 59.9(51.7-67.6) |  |
| Physical activity |  |  |  |  |  | 0.118 |
| No activity, % | 27.4(23.8-31.3) | 31.4(26.8-36.5) | 32.8(27.9-38.0) | 34.3(28.6-40.5) | 33.1(29.1-37.4) |  |
| Moderate activity, % | 32.4(28.0-37.2) | 32.0(28.3-36.0) | 36.0(32.1-40.1) | 30.0(25.6-34.7) | 29.1(24.8-33.8) |  |
| Vigorous activity, % | 40.2(35.4-45.2) | 36.5(32.1-41.3) | 31.2(26.9-35.9) | 35.8(31.4-40.4) | 37.8(32.9-43.0) |  |
| Prevalent hypertension, % | 32.8(28.1-37.9) | 29.1(23.4-35.6) | 32.7(27.9-37.8) | 29.5(25.9-33.3) | 25.8(21.3-30.8) | 0.187 |
| Prevalent cardiovascular, % | 9.5(7.2-12.6) | 6.8(4.9-9.3) | 11.6(9.1-14.7) | 8.4(5.8-12.0) | 8.3(6-11.4) | 0.067 |
| Prevalent cancer, % | 7.2(5.4-9.7) | 11.5(8.2-15.8) | 9.6(6.5-13.9) | 10.1(6.6-15.4) | 7.6(5.4-10.6) | 0.284 |

^‡^Values are weighted means (95% CI) or weighted percentages (95% CI).

^*^*P* <0.05. *P* was assessed with General linear models adjusting for age (continuous variables) or χ^2^ test (bivariate relationships).

All data analyses conducted in the current study were based on estimates with sample weights provided by NHANES.

Table S11. Baseline characteristics according to quintiles of serum docosatetraenoic acid (DTA)^‡^

|  | Serum docosatetraenoic acid quintile (%) | | | | | *p*-trend |
| --- | --- | --- | --- | --- | --- | --- |
|  | Q1(<0.18) | Q2(0.18-0.21) | Q3(0.21-0.24) | Q4(0.24-0.28) | Q5(>0.28) |  |
| Age, years | 50.6(48.5-52.6) | 46.6(44.9-48.4) | 44.2(42.1-46.3) | 44.2(42.4-46.0) | 44.9(43.6-46.1) | <0.001 |
| Male, % | 40.5(35.5-45.8) | 48.4(44.6-52.4) | 48.1(42.3-53.9) | 49.2(44.1-54.3) | 53.8(49.1-58.4) | 0.020 |
| Non-Hispanic white | 71.4(66.3-76.1) | 71.9(64.4-78.4) | 70.4(64.0-76.1) | 70.6(64.0-76.3) | 62.2(52.8-70.8) | 0.024 |
| BMI, kg/m^2^ | 27.0(26.2-27.8) | 28.7(28.0-29.4) | 28.2(27.5-28.8) | 29.1(28.5-29.7) | 28.5(27.8-29.2) | 0.003 |
| WAIST, cm | 94.8(92.5-97.1) | 99(97.4-100.6) | 97.1(95.6-98.6) | 98.8(97.1-100.5) | 97.5(95.8-99.2) | 0.011 |
| Education |  |  |  |  |  | <0.001 |
| Less than high school, % | 10.18(7.52-13.65) | 16.9(13.7-20.7) | 18.2(14.7-22.3) | 20.0(15.2-25.8) | 24.2(20.5-28.3) |  |
| High school or equivalent, % | 16.0(11.9-21.0) | 23.1(18.1-29.0) | 24.5(20.2-29.2) | 24.6(21.0-28.6) | 28.3(23.7-33.4) |  |
| College graduate or above, % | 73.8(67.8-79.1) | 60.0(54.1-65.5) | 57.3(51.0-63.5) | 55.4(50.0-60.7) | 47.5(42.1-53.0) |  |
| Family income-to-poverty ratio |  |  |  |  |  | <0.001 |
| ≤1.0 | 7.73(5.9-10.06) | 13.4(10.3-17.2) | 15.8(12.2-20.2) | 18.7(14.6-23.5) | 21.0(16.6-26.2) |  |
| 1.0-3.0 | 28.4(22.9-34.6) | 35.5(31.2-40.1) | 38.9(33.5-44.6) | 37.0(32.6-41.6) | 42.0(37.2-46.9) |  |
| >3.0 | 63.9(56.9-70.3) | 51.1(45.5-56.6) | 45.2(38.8-51.8) | 44.3(38.2-50.6) | 37.0(31.4-43.0) |  |
| Smoking status |  |  |  |  |  | <0.001 |
| Never smoker, % | 60.3(55.1-65.4) | 53.4(47.4-59.2) | 55.6(51.7-59.5) | 51.9(46.6-57.2) | 48.9(43.5-54.2) |  |
| Former smoker, % | 28.6(25.0-32.4) | 27.4(22.8-32.5) | 23.4(20.1-27) | 18.6(15.3-22.4) | 19.8(15.7-24.8) |  |
| Current smoker, % | 11.09(7.92-15.33) | 19.2(14.6-25.0) | 21.0(17.6-25) | 29.5(24.6-35.0) | 31.3(26.4-36.7) |  |
| Drinking status |  |  |  |  |  | 0.109 |
| Nondrinker, % | 26.2(20.6-32.6) | 30.6(24.0-38.0) | 24.8(19.8-30.7) | 27.2(21.7-33.5) | 29.3(24.8-34.3) |  |
| Low-to-moderate drinker, % | 14.7(11.6-18.4) | 7.5(5.4-10.2) | 9.1(6.3-12.9) | 10.3(7.3-14.2) | 9.6(6.5-13.8) |  |
| Heavy drinker, % | 59.2(52.7-65.4) | 62.0(55.6-68.0) | 66.1(60.2-71.5) | 62.5(56.6-68.1) | 61.1(55.9-66.2) |  |
| Physical activity |  |  |  |  |  | 0.062 |
| No activity, % | 27.0(23.1-31.2) | 31.5(26.5-37.0) | 32.6(28.1-37.5) | 28.3(24.2-32.9) | 37.9(32.9-43.2) |  |
| Moderate activity, % | 33.8(28.9-39.1) | 32.5(27.8-37.6) | 30.8(25.9-36.2) | 36.8(32.8-41.0) | 28.0(24.1-32.2) |  |
| Vigorous activity, % | 39.2(33.3-45.4) | 36.0(31.6-40.5) | 36.6(32.0-41.5) | 34.9(31.4-38.4) | 34.1(29.0-39.5) |  |
| Prevalent hypertension, % | 30.8(25.5-36.6) | 29.5(24.9-34.6) | 28.9(25.1-32.9) | 31.2(26.4-36.5) | 33.8(29.7-38.2) | 0.551 |
| Prevalent cardiovascular, % | 8.2(5.6-12.0) | 9.1(6.4-12.9) | 6.7(4.9-9.1) | 10.5(7.9-13.7) | 10.8(8.4-13.8) | 0.170 |
| Prevalent cancer, % | 12.2(9.0-16.4) | 7.8(5.6-10.8) | 8.6(6.6-11.1) | 8.3(5.5-12.4) | 7.4(5.4-10) | 0.133 |

^‡^Values are weighted means (95% CI) or weighted percentages (95% CI).

^*^*P* <0.05. *P* was assessed with General linear models adjusting for age (continuous variables) or χ^2^ test (bivariate relationships).

All data analyses conducted in the current study were based on estimates with sample weights provided by NHANES.

Table S12. Baseline characteristics according to quintiles of serum n-6 Docosapentaenoic acid (DPAn6)^‡^

|  | Serum n-6 Docosapentaenoic acid quintile (%) | | | | | *p*-trend |
| --- | --- | --- | --- | --- | --- | --- |
|  | Q1(<0.13) | Q2(0.13-0.16) | Q3(0.16-0.19) | Q4(0.19-0.23) | Q5(>0.23) |  |
| Age, years | 49.7(47.7-51.6) | 46.4(44.8-47.9) | 45.8(44.3-47.3) | 43.0(41.2-44.8) | 44.3(42.7-45.9) | <0.001 |
| Male, % | 53.9(49.0-58.8) | 51.8(47.3-56.2) | 49.6(43.8-55.4) | 47.3(42.2-52.4) | 32.9(28.6-37.6) | <0.001 |
| Non-Hispanic white | 76.3(71.4-80.6) | 74.1(67.1-80.0) | 70.5(64.1-76.2) | 65.7(58.3-72.4) | 57.2(49.2-64.9) | <0.001 |
| BMI, kg/m^2^ | 28.3(27.6-29.1) | 28.6(28.0-29.2) | 27.8(27.4-28.3) | 28.3(27.6-29.0) | 28.4(27.7-29.0) | 0.934 |
| WAIST, cm | 98.7(96.7-100.6) | 98.8(97.3-100.4) | 96.7(95.5-97.8) | 96.8(95.0-98.5) | 95.9(94.1-97.7) | 0.114 |
| Education |  |  |  |  |  | <0.001 |
| Less than high school, % | 9.91(7.45-13.06) | 14.1(10.6-18.4) | 21.2(17.3-25.8) | 21.4(17.5-26.0) | 24.3(19.4-30.0) |  |
| High school or equivalent, % | 20.7(17.0-24.9) | 22.8(18.3-27.9) | 23.5(20.3-27.0) | 25.0(20.6-29.9) | 24.5(21.1-28.2) |  |
| College graduate or above, % | 69.4(63.9-74.4) | 63.1(55.9-69.8) | 55.2(51.0-59.4) | 53.6(48.1-59.0) | 51.2(45.7-56.6) |  |
| Family income-to-poverty ratio |  |  |  |  |  | <0.001 |
| ≤1.0 | 8.8(6.1-12.6) | 11.6(9.7-13.9) | 13.9(10.9-17.6) | 23.3(18.3-29.3) | 20.6(16.3-25.7) |  |
| 1.0-3.0 | 28.2(22.7-34.4) | 34.5(29.7-39.7) | 39.4(33.9-45.2) | 39.7(34.2-45.4) | 39.9(34.4-45.6) |  |
| >3.0 | 63.0(55.0-70.4) | 53.8(48.4-59.3) | 46.7(41.8-51.6) | 37.0(30.4-44.1) | 39.5(34.4-44.9) |  |
| Smoking status |  |  |  |  |  | <0.001 |
| Never smoker, % | 56.9(52.9-60.9) | 53.2(47.4-59.0) | 54.6(49.6-59.6) | 53.1(48.1-58.1) | 53.4(48.4-58.3) |  |
| Former smoker, % | 30.3(26.2-34.6) | 22.9(18.9-27.5) | 22.3(18.3-26.8) | 21.4(18.2-24.9) | 19.6(15.5-24.4) |  |
| Current smoker, % | 12.8(9.13-17.67) | 23.9(20.3-27.9) | 23.1(19.2-27.6) | 25.5(21.6-29.9) | 27.0(22.1-32.6) |  |
| Drinking status |  |  |  |  |  | 0.004 |
| Nondrinker, % | 25.7(19.6-33.0) | 26.3(20.3-33.3) | 29.0(24.1-34.5) | 28.0(23.0-33.6) | 29.6(25.2-34.5) |  |
| Low-to-moderate drinker, % | 15.4(12.6-18.7) | 10.3(7.9-13.3) | 9.9(7.2-13.4) | 8.1(6.3-10.4) | 5.1(3.1-8.2) |  |
| Heavy drinker, % | 58.9(52.6-64.9) | 63.4(57.1-69.2) | 61.1(55.8-66.1) | 63.9(58-69.4) | 65.3(60.7-69.7) |  |
| Physical activity |  |  |  |  |  | 0.002 |
| No activity, % | 25.2(20.3-30.7) | 30.2(26.2-34.6) | 31.1(26.6-36.0) | 30.8(26.3-35.6) | 41.0(36.7-45.5) |  |
| Moderate activity, % | 36.5(31.9-41.2) | 29.4(25.1-34.1) | 35.6(30.7-40.9) | 32.4(29.2-35.9) | 29.0(25.1-33.3) |  |
| Vigorous activity, % | 38.4(33.9-43.1) | 40.4(35.4-45.6) | 33.2(28.6-38.3) | 36.8(32.6-41.2) | 30.0(24.5-36.1) |  |
| Prevalent hypertension, % | 34.6(29.9-39.7) | 29.3(25.4-33.5) | 29.8(26.0-33.9) | 28.1(23.6-33.0) | 31.5(28.3-34.7) | 0.093 |
| Prevalent cardiovascular, % | 9.3(6.2-13.6) | 9.0(6.7-11.9) | 8.6(6.7-10.9) | 9.2(6.7-12.6) | 8.7(6.8-11.0) | 0.981 |
| Prevalent cancer, % | 10.0(7.7-12.9) | 10.2(7.5-13.9) | 7.3(4.8-11.0) | 7.8(5.8-10.5) | 9.3(6.3-13.6) | 0.470 |

^‡^Values are weighted means (95% CI) or weighted percentages (95% CI).

^*^*P* <0.05. *P* was assessed with General linear models adjusting for age (continuous variables) or χ^2^ test (bivariate relationships).

All data analyses conducted in the current study were based on estimates with sample weights provided by NHANES.

Table S13. Baseline characteristics according to quintiles of serum palmitoleic acid (PA)^‡^

|  | Serum palmitoleic acid quintile (%) | | | | | *p*-trend |
| --- | --- | --- | --- | --- | --- | --- |
|  | Q1(<1.24) | Q2(1.24-1.61) | Q3(1.61-2.02) | Q4(2.02-2.58) | Q5(>2.58) |  |
| Age, years | 43.5(41.5-45.5) | 45.4(43.4-47.3) | 44.6(42.8-46.3) | 47.7(46.0-49.3) | 49.0(47.3-50.7) | 0.001 |
| Male, % | 60.0(55.1-64.7) | 47.4(43.4-51.4) | 47.8(43.8-51.8) | 43.3(38.9-47.8) | 43.0(37.1-49.2) | <0.001 |
| Non-Hispanic white | 58.0(49.3-66.1) | 66.0(58.6-72.7) | 71.1(65.6-76.0) | 75.6(70.3-80.3) | 75.0(68.6-80.4) | <0.001 |
| BMI, kg/m^2^ | 26.5(26.0-27.0) | 27.4(26.9-28.0) | 27.7(27.2-28.3) | 29.6(28.9-30.3) | 29.9(29.3-30.5) | <0.001 |
| WAIST, cm | 92.7(91.3-94.1) | 95.3(93.9-96.8) | 95.6(94-97.1) | 101(99.3-102.7) | 102.1(100.3-103.8) | <0.001 |
| Education |  |  |  |  |  | 0.164 |
| Less than high school, % | 15.5(11.9-20.0) | 17.1(13.8-21.0) | 18.1(13.4-23.9) | 17.7(14.3-21.6) | 20.8(17.5-24.6) |  |
| High school or equivalent, % | 21.1(16.5-26.6) | 22.8(19.2-26.9) | 20.7(16.4-25.7) | 25.6(20.6-31.3) | 25.7(20.5-31.6) |  |
| College graduate or above, % | 63.4(56.6-69.6) | 60.1(55.2-64.8) | 61.3(56.5-65.8) | 56.8(50.4-63.0) | 53.5(47.9-59.0) |  |
| Family income-to-poverty ratio |  |  |  |  |  | 0.051 |
| ≤1.0 | 15.6(12.5-19.3) | 15.6(12.3-19.6) | 15.3(11.9-19.5) | 15.0(11.7-19.0) | 15.5(12.6-18.8) |  |
| 1.0-3.0 | 32.1(27.3-37.3) | 38.7(32.8-45.0) | 32.7(27.4-38.5) | 35.2(31.0-39.5) | 42.2(36.1-48.6) |  |
| >3.0 | 52.3(46.5-58.1) | 45.6(39.9-51.5) | 51.9(46.1-57.8) | 49.9(44.4-55.4) | 42.3(35.5-49.5) |  |
| Smoking status |  |  |  |  |  | <0.001 |
| Never smoker, % | 61.4(55.9-66.6) | 60.6(56.2-64.8) | 58.2(52.7-63.6) | 51.6(46.1-57.2) | 39.9(34.3-45.8) |  |
| Former smoker, % | 25.8(23.0-28.8) | 21.6(17.4-26.4) | 20.1(16.3-24.6) | 22.8(18.9-27.1) | 28.5(24.2-33.1) |  |
| Current smoker, % | 12.83(9.15-17.71) | 17.8(14.9-21.1) | 21.7(17.2-26.9) | 25.6(20.2-31.9) | 31.6(27.0-36.7) |  |
| Drinking status |  |  |  |  |  | <0.001 |
| Nondrinker, % | 29.4(24.5-34.7) | 29.7(23.3-36.9) | 27(22.3-32.3) | 28.2(23.3-33.7) | 24.3(20.1-29.0) |  |
| Low-to-moderate drinker, % | 17.8(13.7-22.8) | 13.8(10.8-17.5) | 8.4(6.5-10.7) | 7.3(4.8-10.9) | 4.2(2.7-6.5) |  |
| Heavy drinker, % | 52.8(47.5-58.1) | 56.5(49.6-63.3) | 64.6(58.9-69.9) | 64.5(59.1-69.6) | 71.5(66.5-76.0) |  |
| Physical activity |  |  |  |  |  | <0.001 |
| No activity, % | 24.1(20.2-28.6) | 30.5(26.5-34.8) | 32.0(27.6-36.8) | 32.5(27.7-37.7) | 37.4(32.3-42.8) |  |
| Moderate activity, % | 32.7(27.6-38.2) | 31.4(27.6-35.6) | 30.9(26.5-35.7) | 31.9(27.9-36.2) | 36.3(32.5-40.3) |  |
| Vigorous activity, % | 43.1(37.6-48.8) | 38.1(32.7-43.8) | 37.0(32.5-41.7) | 35.6(30.0-41.7) | 26.3(22.6-30.3) |  |
| Prevalent hypertension, % | 23.1(19.7-26.9) | 26.1(21.5-31.4) | 28.5(24.0-33.6) | 34.2(28.3-40.7) | 41.6(36.6-46.8) | <0.001 |
| Prevalent cardiovascular, % | 4.9(3.4-7.2) | 7.4(5.8-9.5) | 8.8(6.3-12.3) | 11.2(8.3-14.8) | 12.5(9.4-16.4) | <0.001 |
| Prevalent cancer, % | 8.8(5.7-13.3) | 8.3(5.8-11.9) | 8.6(6.4-11.5) | 10.3(7.8-13.5) | 9.8(6.6-14.4) | 0.853 |

^‡^Values are weighted means (95% CI) or weighted percentages (95% CI).

^*^*P* <0.05. *P* was assessed with General linear models adjusting for age (continuous variables) or χ^2^ test (bivariate relationships).

All data analyses conducted in the current study were based on estimates with sample weights provided by NHANES.

Table S14. Baseline characteristics according to quintiles of serum cis-Vaccenic acid (VA)^‡^

|  | Serum cis-Vaccenic acid quintile (%) | | | | | *p*-trend |
| --- | --- | --- | --- | --- | --- | --- |
|  | Q1(<1.08) | Q2(1.08-1.21) | Q3(1.21-1.34) | Q4(1.34-1.5) | Q5(>1.5) |  |
| Age, years | 42.4(40.6-44.1) | 44.3(42.8-45.8) | 45.5(43.9-47.0) | 47.8(45.8-49.7) | 51.3(49.2-53.5) | <0.001 |
| Male, % | 43.0(37.8-48.4) | 50.6(46.8-54.3) | 50.8(45.0-56.6) | 47.2(42.3-52.1) | 48.8(44.5-53.0) | 0.163 |
| Non-Hispanic white | 68.1(60.9-74.5) | 68.7(62.4-74.4) | 70.4(63.1-76.8) | 70.8(63.4-77.3) | 69.5(64.1-74.4) | 0.809 |
| BMI, kg/m^2^ | 28.2(27.4-28.9) | 28.2(27.6-28.8) | 28.1(27.5-28.7) | 28.4(27.6-29.1) | 28.5(28.0-29.0) | 0.884 |
| WAIST, cm | 96.4(94.5-98.2) | 97.5(95.6-99.4) | 97.2(95.8-98.6) | 97.4(95.5-99.2) | 98.8(97.1-100.5) | 0.879 |
| Education |  |  |  |  |  | 0.011 |
| Less than high school, % | 13.0(10.0-16.8) | 16.4(13.5-19.9) | 16.7(12.1-22.4) | 20.4(15.7-26.0) | 24.4(20.2-29.0) |  |
| High school or equivalent, % | 23.2(18.2-29.0) | 21.7(17.2-27.0) | 24.7(19.9-30.1) | 22.8(18.4-28.0) | 22.8(18.7-27.5) |  |
| College graduate or above, % | 63.8(57.1-70.0) | 61.8(55.7-67.6) | 58.7(54.1-63.1) | 56.8(50.3-63.0) | 52.8(47.5-58.1) |  |
| Family income-to-poverty ratio |  |  |  |  |  | 0.056 |
| ≤1.0 | 14.8(10.8-20.0) | 14.9(12.1-18.3) | 16.3(13.0-20.2) | 14.9(11.8-18.6) | 16.9(13.6-20.9) |  |
| 1.0-3.0 | 32.5(27.3-38.2) | 32.1(27.0-37.6) | 36.5(30.5-43.0) | 38.9(33.1-45.0) | 41.5(35.2-48.1) |  |
| >3.0 | 52.7(46.9-58.5) | 53.0(46.9-59.1) | 47.2(40.7-53.8) | 46.2(39.5-53.1) | 41.6(35.7-47.7) |  |
| Smoking status |  |  |  |  |  | 0.030 |
| Never smoker, % | 61.4(56.9-65.6) | 53.9(48.8-58.9) | 50.9(43.8-57.9) | 53.2(48.7-57.6) | 50.4(44.1-56.6) |  |
| Former smoker, % | 19.9(16.2-24.2) | 23.2(19.9-27.0) | 23.0(19.0-27.5) | 27.6(23.1-32.7) | 24.8(20.4-29.8) |  |
| Current smoker, % | 18.8(15.3-22.9) | 22.9(18.2-28.4) | 26.1(20.7-32.3) | 19.2(15.4-23.8) | 24.8(20.7-29.5) |  |
| Drinking status |  |  |  |  |  | 0.234 |
| Nondrinker, % | 24.7(20.5-29.5) | 27.4(22.6-32.8) | 25.6(20.8-31.1) | 27.8(21.9-34.6) | 32.5(25.9-39.8) |  |
| Low-to-moderate drinker, % | 8.3(6.0-11.3) | 9.3(6.7-12.8) | 11.2(8.8-14.1) | 11.6(8.3-15.8) | 9.1(6.7-12.2) |  |
| Heavy drinker, % | 67.1(61.6-72.1) | 63.3(57.4-68.9) | 63.2(58.1-68.0) | 60.6(54.9-66.1) | 58.4(51.3-65.2) |  |
| Physical activity |  |  |  |  |  | <0.001 |
| No activity, % | 26.2(22.1-30.8) | 27.1(23.7-30.8) | 31.0(25.1-37.5) | 32.9(28.7-37.4) | 41.2(35.8-46.8) |  |
| Moderate activity, % | 34.0(30.3-37.9) | 32.3(28.0-36.9) | 29.3(25.0-34.0) | 35.3(30.6-40.2) | 31.7(27.0-36.8) |  |
| Vigorous activity, % | 39.8(34.3-45.5) | 40.6(35.3-46.1) | 39.7(34.5-45.1) | 31.8(26.9-37.2) | 27.1(21.5-33.5) |  |
| Prevalent hypertension, % | 22.4(18.3-27.1) | 28.1(23.6-33.2) | 28.3(23.4-33.7) | 32.6(27.6-38.2) | 43.8(37.2-50.6) | <0.001 |
| Prevalent cardiovascular, % | 6.1(3.7-10.1) | 6.8(4.6-9.9) | 6.4(4.4-9.1) | 12.0(9.5-15.2) | 14.9(12.1-18.3) | <0.001 |
| Prevalent cancer, % | 8.2(5.5-11.9) | 8.5(5.9-12.1) | 9.5(6.9-13) | 7.1(5-9.8) | 13.2(10.2-17.0) | 0.054 |

^‡^Values are weighted means (95% CI) or weighted percentages (95% CI).

^*^*P* <0.05. *P* was assessed with General linear models adjusting for age (continuous variables) or χ^2^ test (bivariate relationships).

All data analyses conducted in the current study were based on estimates with sample weights provided by NHANES.

Table S15. Baseline characteristics according to quintiles of serum Oleic acid (OA)^‡^

|  | Serum Oleic acid quintile (%) | | | | | *p*-trend |
| --- | --- | --- | --- | --- | --- | --- |
|  | Q1(<15.96) | Q2(15.96-17.51) | Q3(17.51-19.13) | Q4(19.13-20.97) | Q5(>20.97) |  |
| Age, years | 39.7(37.5-41.9) | 44.5(42.9-46.1) | 45.7(43.7-47.6) | 47.5(45.7-49.3) | 52.3(50.9-53.6) | <0.001 |
| Male, % | 34.8(31.3-38.5) | 42.3(37.4-47.3) | 45.0(40.7-49.3) | 55.3(50.2-60.3) | 61.1(56.4-65.6) | <0.001 |
| Non-Hispanic white | 52.6(44.1-61.0) | 67.8(60.9-74.0) | 73.9(68.0-79.0) | 75.4(69.6-80.3) | 75.9(70.0-80.9) | <0.001 |
| BMI, kg/m^2^ | 27.1(26.5-27.7) | 27.6(27.1-28.2) | 28.0(27.3-28.7) | 29.4(28.5-30.2) | 29.4(28.8-29.9) | <0.001 |
| WAIST, cm | 91.9(90.5-93.3) | 94.5(93.2-95.9) | 96.9(95.1-98.7) | 100.9(98.8-102.9) | 102.9(101.5-104.4) | <0.001 |
| Education |  |  |  |  |  | 0.037 |
| Less than high school, % | 15.2(12.2-18.9) | 17.9(13.2-23.7) | 17.7(13.9-22.3) | 19.3(15.6-23.6) | 19.0(15.5-23.2) |  |
| High school or equivalent, % | 22.1(18.3-26.4) | 18.9(15.2-23.3) | 25.8(21.4-30.8) | 26.3(21.9-31.2) | 22.9(19.9-26.2) |  |
| College graduate or above, % | 62.7(57.2-67.8) | 63.2(57.2-68.9) | 56.5(50.7-62.1) | 54.4(49.1-59.6) | 58.0(53.5-62.5) |  |
| Family income-to-poverty ratio |  |  |  |  |  | 0.272 |
| ≤1.0 | 17.0(12.7-22.4) | 18.6(14.6-23.5) | 14.4(11.5-17.9) | 13.6(11.2-16.4) | 13.5(10.1-17.9) |  |
| 1.0-3.0 | 35.2(29.1-41.8) | 36.9(32.3-41.8) | 33.6(28.4-39.2) | 36.8(31.5-42.3) | 38.5(32.3-45.2) |  |
| >3.0 | 47.8(41.5-54.3) | 44.5(39.2-49.9) | 52.0(44.9-59.0) | 49.7(43.9-55.5) | 48.0(41.5-54.5) |  |
| Smoking status |  |  |  |  |  | <0.001 |
| Never smoker, % | 67.4(62.5-71.9) | 59.3(54.3-64.1) | 55.5(51.3-59.7) | 48.2(42.4-54.0) | 41.3(35.7-47.2) |  |
| Former smoker, % | 18.8(15.8-22.3) | 21.0(17.3-25.1) | 22.4(18.2-27.4) | 26.4(22.5-30.7) | 29.1(24.4-34.3) |  |
| Current smoker, % | 13.8(9.8-19.2) | 19.8(15.0-25.6) | 22.0(17.0-28.0) | 25.5(20.8-30.7) | 29.5(25.8-33.6) |  |
| Drinking status |  |  |  |  |  | 0.002 |
| Nondrinker, % | 25.8(21.8-30.4) | 28.1(23.1-33.6) | 23.0(18.8-27.8) | 26.0(20.3-32.8) | 35.1(28.8-41.9) |  |
| Low-to-moderate drinker, % | 8.2(6.0-11.1) | 9.7(7.1-13.3) | 8.2(6.3-10.7) | 11.1(8.4-14.6) | 12.2(9.4-15.8) |  |
| Heavy drinker, % | 66.0(61.1-70.5) | 62.2(56.6-67.5) | 68.8(64.9-72.4) | 62.9(57.5-67.9) | 52.7(46.2-59.2) |  |
| Physical activity |  |  |  |  |  | 0.002 |
| No activity, % | 27.7(24.3-31.3) | 28.9(25.1-33.2) | 31.2(26.9-35.9) | 31.4(27.5-35.6) | 38.4(33.0-44.1) |  |
| Moderate activity, % | 30.1(25.1-35.6) | 32.0(27.9-36.4) | 32.8(29.3-36.6) | 31.7(27.6-36.1) | 36.1(31.7-40.7) |  |
| Vigorous activity, % | 42.2(35.9-48.9) | 39.0(34.1-44.3) | 36.0(31.4-40.7) | 36.9(32.4-41.7) | 25.6(22.2-29.3) |  |
| Prevalent hypertension, % | 21.5(17.7-25.7) | 27.0(22.1-32.5) | 25.7(22.1-29.6) | 33.6(28.6-39.1) | 45.8(40.7-51.0) | <0.001 |
| Prevalent cardiovascular, % | 2.7(1.9-3.8) | 8.2(6-11) | 6.7(4.4-10.0) | 8.8(6.1-12.5) | 18.8(15.5-22.6) | <0.001 |
| Prevalent cancer, % | 7.7(4.8-12.3) | 7.2(5-10.2) | 9.1(7.1-11.7) | 9.7(7.6-12.3) | 11.8(9.8-14.1) | 0.124 |

^‡^Values are weighted means (95% CI) or weighted percentages (95% CI).

^*^*P* <0.05. *P* was assessed with General linear models adjusting for age (continuous variables) or χ^2^ test (bivariate relationships).

All data analyses conducted in the current study were based on estimates with sample weights provided by NHANES.

Table S16. Baseline characteristics according to quintiles of serum eicosenoic acid (EA)^‡^

|  | Serum eicosenoic acid quintile (%) | | | | | *p*-trend |
| --- | --- | --- | --- | --- | --- | --- |
|  | Q1(<0.1) | Q2(0.1-0.11) | Q3(0.11-0.13) | Q4(0.13-0.15) | Q5(>0.15) |  |
| Age, years | 42.7(41.1-44.4) | 44.2(42.4-45.9) | 47.2(45.2-49.2) | 47.2(45.3-49.2) | 48.5(46.6-50.3) | <0.001 |
| Male, % | 37.6(32.5-43.0) | 46.3(41.7-50.9) | 47.0(43.0-51.1) | 54.2(49.9-58.4) | 56.7(51.4-61.8) | <0.001 |
| Non-Hispanic white | 66.4(58.1-73.7) | 67.9(61.3-73.9) | 73.2(67.6-78.1) | 74.0(67.6-79.5) | 65.9(59.3-71.9) | 0.009 |
| BMI, kg/m^2^ | 29.0(28.3-29.7) | 28.3(27.4-29.1) | 28.3(27.6-29.0) | 28.1(27.5-28.7) | 27.9(27.2-28.6) | 0.017 |
| WAIST, cm | 97.6(95.8-99.3) | 97.0(95.1-98.9) | 97.1(95.4-98.7) | 97.9(96.4-99.4) | 98.1(96-100.2) | 0.782 |
| Education |  |  |  |  |  | 0.330 |
| Less than high school, % | 13.9(11.0-17.5) | 19.3(14.6-24.9) | 17.2(13.4-21.9) | 20.5(16.0-25.8) | 19.1(15.6-23.2) |  |
| High school or equivalent, % | 25.1(20.4-30.3) | 24.8(18.5-32.5) | 23.4(20.4-26.7) | 21.8(17.2-27.2) | 21.3(17.9-25.1) |  |
| College graduate or above, % | 61.0(54.5-67.2) | 55.9(47.7-63.8) | 59.4(55.3-63.4) | 57.7(51.3-63.9) | 59.6(55.3-63.8) |  |
| Family income-to-poverty ratio |  |  |  |  |  | 0.347 |
| ≤1.0 | 17.0(13.0-22.0) | 16.4(13.3-20.0) | 15.2(11.4-20.0) | 13.9(11.9-16.3) | 13.1(9.86-17.2) |  |
| 1.0-3.0 | 35.0(28.9-41.7) | 33.4(28.6-38.6) | 39.1(34.0-44.4) | 34.1(28.4-40.3) | 39.4(33.4-45.8) |  |
| >3.0 | 47.9(41.6-54.3) | 50.2(43.2-57.3) | 45.7(39.6-52.0) | 52.0(45.9-57.9) | 47.5(41.3-53.7) |  |
| Smoking status |  |  |  |  |  | 0.210 |
| Never smoker, % | 58.2(52.9-63.4) | 52.0(47.3-56.8) | 56.0(50.4-61.3) | 52.7(47.1-58.2) | 50.9(47.0-54.8) |  |
| Former smoker, % | 19.5(16.2-23.2) | 23.2(18.5-28.5) | 20.9(16.8-25.7) | 25.3(21.0-30.2) | 28.3(23.4-33.9) |  |
| Current smoker, % | 22.3(18.7-26.4) | 24.8(19.2-31.5) | 23.1(18.5-28.5) | 22.0(17.6-27.2) | 20.8(16.1-26.4) |  |
| Drinking status |  |  |  |  |  | 0.010 |
| Nondrinker, % | 25.6(22.6-28.8) | 24.2(18.6-30.8) | 31.1(24.5-38.6) | 28.9(23.1-35.5) | 29.6(23.7-36.2) |  |
| Low-to-moderate drinker, % | 6.5(4.4-9.3) | 8.9(6.3-12.5) | 9.6(7.1-13.0) | 12.4(9.9-15.3) | 13.2(10.0-17.2) |  |
| Heavy drinker, % | 68.0(64.1-71.6) | 66.9(61.1-72.2) | 59.3(52.1-66.1) | 58.7(53.6-63.7) | 57.3(50.7-63.6) |  |
| Physical activity |  |  |  |  |  | 0.443 |
| No activity, % | 28.9(25.1-33.0) | 30.1(26.7-33.7) | 32.1(27.5-37.1) | 33.5(27.6-39.9) | 33.3(29.3-37.6) |  |
| Moderate activity, % | 33.9(30.1-38.0) | 31.7(27.7-36.1) | 32.2(27.0-37.9) | 28.3(24.1-33.0) | 34.9(30.1-40.1) |  |
| Vigorous activity, % | 37.2(31.8-42.9) | 38.2(33.4-43.2) | 35.7(31.2-40.5) | 38.2(32.4-44.3) | 31.8(25.5-38.8) |  |
| Prevalent hypertension, % | 25.5(20.8-30.8) | 29.1(25.6-33.0) | 27.3(23.4-31.5) | 37.1(31.4-43.2) | 32.3(27.5-37.5) | 0.003 |
| Prevalent cardiovascular, % | 6.1(4.1-9.0) | 7.3(5.1-10.5) | 7.5(5.3-10.4) | 10.7(8.2-13.8) | 14.0(10.7-18.1) | <0.001 |
| Prevalent cancer, % | 8.1(4.9-13.2) | 6.9(4.8-9.8) | 10.2(6.7-15.2) | 9.9(7.1-13.5) | 10.4(8.0-13.6) | 0.493 |

^‡^Values are weighted means (95% CI) or weighted percentages (95% CI).

^*^*P* <0.05. *P* was assessed with General linear models adjusting for age (continuous variables) or χ^2^ test (bivariate relationships).

All data analyses conducted in the current study were based on estimates with sample weights provided by NHANES.

Table S17. Baseline characteristics according to quintiles of serum Nervonic acid (NRA)^‡^

|  | Serum Nervonic acid quintile (%) | | | | | *p*-trend |
| --- | --- | --- | --- | --- | --- | --- |
|  | Q1(<0.56) | Q2(0.56-0.68) | Q3(0.68-0.79) | Q4(0.79-0.92) | Q5(>0.92) |  |
| Age, years | 47.1(45.8-48.5) | 45.3(43.6-47.0) | 45.3(43.7-46.9) | 44.3(42.3-46.3) | 47.2(45.1-49.2) | 0.466 |
| Male, % | 60.8(56.4-65.1) | 49.5(45.8-53.2) | 45.6(41.6-49.7) | 45.6(39.4-51.9) | 37.1(32.3-42.1) | <0.001 |
| Non-Hispanic white | 73.9(66.8-79.9) | 73.0(65.0-79.7) | 70.9(64.4-76.7) | 64.9(57.2-71.9) | 60.6(53.8-67.1) | <0.001 |
| BMI, kg/m^2^ | 30.0(29.4-30.6) | 28.5(28.0-28.9) | 28.0(27.4-28.5) | 27.5(26.8-28.2) | 27.2(26.3-28.1) | <0.001 |
| WAIST, cm | 103.4(101.5-105.2) | 98(96.7-99.3) | 96.5(95.2-97.9) | 94.7(93.1-96.4) | 93.1(91.0-95.2) | <0.001 |
| Education |  |  |  |  |  | 0.759 |
| Less than high school, % | 18.1(13.6-23.6) | 18.9(14.5-24.2) | 17.2(14.0-20.9) | 17.3(12.9-22.9) | 17.1(12.9-22.3) |  |
| High school or equivalent, % | 26.1(22.0-30.6) | 23.6(20.6-26.9) | 22.8(18.8-27.4) | 21.4(16.0-28.1) | 22.7(18.4-27.8) |  |
| College graduate or above, % | 55.9(50.2-61.4) | 57.5(51.8-63.0) | 60.0(56.5-63.4) | 61.2(54.4-67.6) | 60.2(54.2-65.9) |  |
| Family income-to-poverty ratio |  |  |  |  |  | 0.215 |
| ≤1.0 | 15.3(11.4-20.0) | 12.8(9.5-17.0) | 14.2(11.8-17.0) | 15.7(11.9-20.4) | 17.9(13.3-23.8) |  |
| 1.0-3.0 | 30.7(26.6-35.1) | 39.1(33.7-44.7) | 38.3(32.7-44.2) | 36.8(32.0-41.9) | 35.1(27.6-43.4) |  |
| >3.0 | 54.1(49.4-58.6) | 48.1(41.4-54.8) | 47.5(40.9-54.2) | 47.5(41.3-53.8) | 47.0(38.1-56) |  |
| Smoking status |  |  |  |  |  | <0.001 |
| Never smoker, % | 44.2(39.1-49.5) | 55.0(50.8-59.1) | 53.8(50.5-57.2) | 57.3(52.6-61.9) | 59.5(54.1-64.7) |  |
| Former smoker, % | 28.3(23.6-33.5) | 24.6(21.2-28.2) | 23.2(20.2-26.4) | 18.2(15-21.9) | 22.9(19.0-27.3) |  |
| Current smoker, % | 27.4(22.1-33.6) | 20.5(16.5-25.1) | 23.0(19.4-27.0) | 24.5(20.2-29.4) | 17.6(13.0-23.3) |  |
| Drinking status |  |  |  |  |  | 0.282 |
| Nondrinker, % | 31.0(24.7-38.0) | 26.7(21.6-32.5) | 24.2(19.3-30.0) | 28.2(23.1-33.9) | 26.7(22.0--32.0) |  |
| Low-to-moderate drinker, % | 8.7(6.4-11.8) | 8.8(6.1-12.6) | 12.5(10.1-15.3) | 10.9(8.3-14.3) | 9.5(7.1-12.6) |  |
| Heavy drinker, % | 60.3(54.1-66.2) | 64.5(59.7-69.1) | 63.3(58.0-68.3) | 60.8(54.7-66.7) | 63.8(58.7-68.6) |  |
| Physical activity |  |  |  |  |  | 0.068 |
| No activity, % | 30.8(25.6-36.5) | 29.3(25.5-33.5) | 31.8(27.7-36.2) | 28.0(24.1-32.1) | 36.0(30.5-42.0) |  |
| Moderate activity, % | 33.6(28.1-39.7) | 37.3(32.8-42.0) | 30.3(25.6-35.4) | 28.8(25.0-33.0) | 29.2(25.4-33.4) |  |
| Vigorous activity, % | 35.6(30.5-41.0) | 33.4(29.4-37.5) | 38.0(32.1-44.2) | 43.2(37.7-48.9) | 34.7(28.6-41.4) |  |
| Prevalent hypertension, % | 36.5(30.7-42.6) | 35.5(31.5-39.7) | 26.3(21.4-31.8) | 23.5(20-27.4) | 26.7(21.8-32.2) | <0.001 |
| Prevalent cardiovascular, % | 13.5(10.6-16.9) | 8.3(5.5-12.1) | 7.2(5.4-9.4) | 8.5(5.7-12.3) | 7.1(5.4-9.3) | 0.002 |
| Prevalent cancer, % | 7.8(5.8-10.5) | 7.7(5.5-10.7) | 13.1(9.5-17.8) | 8.0(5.7-11.2) | 8.9(6.2-12.6) | 0.044 |

^‡^Values are weighted means (95% CI) or weighted percentages (95% CI).

^*^*P* <0.05. *P* was assessed with General linear models adjusting for age (continuous variables) or χ^2^ test (bivariate relationships).

All data analyses conducted in the current study were based on estimates with sample weights provided by NHANES.

Table S18. Adjusted OR for associations of different n-3 PUFAs with the prevalence of T2DM

|  | T2DM | | | | | | | | | |
| --- | --- | --- | --- | --- | --- | --- | --- | --- | --- | --- |
|  |  | | Model1 | |  | Model2 | |  | Model3 | |
|  | Case/N | OR(95% CI) | | P-value |  | OR(95% CI) | P-value |  | OR(95% CI) | P-value |
| **ALA(n3),%** |  |  | |  |  |  |  |  |  |  |
| Q1(<0.48) | 135/763 | 1(Ref) | |  |  | 1(Ref) |  |  | 1(Ref) |  |
| Q2(0.48-0.58) | 142/765 | 0.905(0.579,1.417) | | 0.653 |  | 1.197(0.727,1.970) | 0.450 |  | 1.24(0.743,2.069) | 0.372 |
| Q3(0.58-0.69) | 134/750 | 1.047(0.706,1.553) | | 0.811 |  | 1.281(0.767,2.139) | 0.316 |  | 1.306(0.787,2.166) | 0.268 |
| Q4(0.69-0.86) | 184/730 | 1.336(0.973,1.833) | | 0.071 |  | 1.500(1.113,2.020) | 0.012 |  | 1.561(1.160,2.100) | 0.007 |
| Q5(>0.86) | 169/742 | 1.476(1.015,2.146) | | 0.042 |  | 1.974(1.245,3.128) | 0.007 |  | 1.891(1.202,2.975) | 0.011 |
| **Per 1SD change** |  | 1.157(1.054,1.270) | | 0.003 |  | 1.201(1.073,1.344) | 0.003 |  | 1.196(1.063,1.346) | 0.006 |
| ***p* for trend** |  |  | | 0.019 |  |  | 0.003 |  |  | 0.004 |
| **EPA(n3),%** |  |  | |  |  |  |  |  |  |  |
| Q1(<0.26) | 134/786 | 1(Ref) | |  |  | 1(Ref) |  |  | 1(Ref) |  |
| Q2(0.26-0.35) | 142/770 | 0.841(0.532,1.329) | | 0.444 |  | 0.765(0.450,1.300) | 0.295 |  | 0.735(0.426,1.268) | 0.237 |
| Q3(0.35-0.44) | 152/743 | 0.759(0.463,1.242) | | 0.260 |  | 0.837(0.482,1.452) | 0.497 |  | 0.840(0.471,1.496) | 0.515 |
| Q4(0.44-0.63) | 175/735 | 0.780(0.520,1.168) | | 0.217 |  | 0.794(0.538,1.170) | 0.220 |  | 0.784(0.510,1.207) | 0.238 |
| Q5(>0.63) | 167/725 | 0.452(0.307,0.663) | | <0.001 |  | 0.625(0.369,1.059) | 0.076 |  | 0.634(0.369,1.088) | 0.089 |
| **Per 1SD change** |  | 0.746(0.634,0.878) | | 0.001 |  | 0.883(0.714,1.092) | 0.231 |  | 0.885(0.720,1.087) | 0.222 |
| ***p* for trend** |  |  | | <0.001 |  |  | 0.088 |  |  | 0.108 |
| **DPAn3(n3),%** |  |  | |  |  |  |  |  |  |  |
| Q1(<0.32) | 158/765 | 1(Ref) | |  |  | 1(Ref) |  |  | 1(Ref) |  |
| Q2(0.32-0.38) | 145/747 | 0.726(0.547,0.965) | | 0.029 |  | 0.837(0.583,1.201) | 0.306 |  | 0.834(0.557,1.249) | 0.339 |
| Q3(0.38-0.43) | 142/740 | 0.640(0.468,0.876) | | 0.007 |  | 0.671(0.431,1.046) | 0.074 |  | 0.682(0.443,1.050) | 0.076 |
| Q4(0.43-0.49) | 155/732 | 0.577(0.390,0.854) | | 0.008 |  | 0.746(0.462,1.205) | 0.209 |  | 0.728(0.440,1.204) | 0.190 |
| Q5(>0.49) | 162/731 | 0.437(0.309,0.616) | | <0.001 |  | 0.598(0.377,0.949) | 0.032 |  | 0.595(0.375,0.942) | 0.030 |
| **Per 1SD change** |  | 0.758(0.671,0.857) | | <0.001 |  | 0.828(0.714,0.959) | 0.015 |  | 0.823(0.714,0.948) | 0.011 |
| ***p* for trend** |  |  | | <0.001 |  |  | 0.037 |  |  | 0.028 |
| **DHA(n3),%** |  |  | |  |  |  |  |  |  |  |
| Q1(<0.87) | 135/755 | 1(Ref) | |  |  | 1(Ref) |  |  | 1(Ref) |  |
| Q2(0.87-1.09) | 157/760 | 0.963(0.672,1.379) | | 0.831 |  | 1.080(0.773,1.509) | 0.627 |  | 1.086(0.755,1.564) | 0.623 |
| Q3(1.09-1.33) | 146/748 | 0.905(0.684,1.199) | | 0.473 |  | 1.081(0.765,1.528) | 0.636 |  | 1.082(0.755,1.548) | 0.637 |
| Q4(1.33-1.72) | 164/752 | 0.879(0.600,1.288) | | 0.494 |  | 1.007(0.667,1.518) | 0.972 |  | 0.973(0.627,1.510) | 0.891 |
| Q5(>1.72) | 165/736 | 0.540(0.401,0.727) | | <0.001 |  | 0.795(0.543,1.166) | 0.218 |  | 0.803(0.539,1.198) | 0.250 |
| **Per 1SD change** |  | 0.806(0.717,0.906) | | 0.001 |  | 0.942(0.786,1.130) | 0.497 |  | 0.939(0.783,1.126) | 0.468 |
| ***p* for trend** |  |  | | <0.001 |  |  | 0.244 |  |  | 0.200 |

Model1: adjusted for age;

Model2: adjusted for age, sex, race, education level, BMI, waist circumference, family income-to-poverty ratio, smoking status, drinking status and physical activity;

Model3: adjusted for age, sex, race, education level, BMI, waist circumference, family income-to-poverty ratio, smoking status, drinking status and physical activity, hypertension, cardiovascular disease and cancer.

Table S19. Adjusted OR for associations of different n-6 PUFAs with the prevalence of T2DM

|  | T2DM | | | | | | | | | |
| --- | --- | --- | --- | --- | --- | --- | --- | --- | --- | --- |
|  |  | | Model1 | |  | Model2 | |  | Model3 | |
|  | Case/N | OR(95% CI) | | P-value |  | OR(95% CI) | P-value |  | OR(95% CI) | P-value |
| **LA(n6),%** |  |  | |  |  |  |  |  |  |  |
| Q1(<27.71) | 264/725 | 1(Ref) | |  |  | 1(Ref) |  |  | 1(Ref) |  |
| Q2(27.71-30.7) | 198/740 | 0.599(0.414,0.866) | | 0.008 |  | 0.881(0.574,1.352) | 0.534 |  | 0.948(0.619,1.452) | 0.786 |
| Q3(30.70-32.96) | 131/750 | 0.388(0.284,0.529) | | <0.001 |  | 0.445(0.307,0.645) | <0.001 |  | 0.457(0.307,0.680) | 0.001 |
| Q4(32.96-35.63) | 115/755 | 0.367(0.221,0.607) | | <0.001 |  | 0.551(0.315,0.961) | 0.038 |  | 0.595(0.326,1.086) | 0.084 |
| Q5(>35.63) | 61/785 | 0.219(0.121,0.398) | | <0.001 |  | 0.259(0.152,0.443) | <0.001 |  | 0.277(0.163,0.472) | <0.001 |
| **Per 1SD change** |  | 0.603(0.523,0.695) | | <0.001 |  | 0.647(0.56,0.747) | <0.001 |  | 0.664(0.572,0.772) | <0.001 |
| ***p* for trend** |  |  | | <0.001 |  |  | <0.001 |  |  | <0.001 |
| **GLA(n6),%** |  |  | |  |  |  |  |  |  |  |
| Q1(<0.3) | 127/778 | 1(Ref) | |  |  | 1(Ref) |  |  | 1(Ref) |  |
| Q2(0.3-0.39) | 148/746 | 1.173(0.746,1.845) | | 0.476 |  | 0.846(0.496,1.443) | 0.511 |  | 0.829(0.474,1.448) | 0.470 |
| Q3(0.39-0.48) | 160/737 | 1.103(0.737,1.650) | | 0.623 |  | 0.783(0.482,1.271) | 0.295 |  | 0.766(0.452,1.299) | 0.287 |
| Q4(0.48-0.61) | 159/725 | 0.894(0.621,1.286) | | 0.531 |  | 0.689(0.440,1.079) | 0.096 |  | 0.667(0.411,1.085) | 0.093 |
| Q5(>0.61) | 163/713 | 0.894(0.598,1.338) | | 0.574 |  | 0.622(0.350,1.104) | 0.097 |  | 0.594(0.318,1.113) | 0.094 |
| **Per 1SD change** |  | 0.931(0.840,1.033) | | 0.170 |  | 0.858(0.713,1.032) | 0.098 |  | 0.847(0.692,1.037) | 0.099 |
| ***p* for trend** |  |  | | 0.169 |  |  | 0.064 |  |  | 0.059 |
| **DGLA(n6),%** |  |  | |  |  |  |  |  |  |  |
| Q1(<1.07) | 184/733 | 1(Ref) | |  |  | 1(Ref) |  |  | 1(Ref) |  |
| Q2(1.07-1.24) | 157/728 | 0.527(0.364,0.764) | | 0.001 |  | 0.519(0.306,0.883) | 0.019 |  | 0.516(0.296,0.899) | 0.024 |
| Q3(1.24-1.4) | 127/720 | 0.522(0.358,0.760) | | 0.001 |  | 0.439(0.258,0.748) | 0.005 |  | 0.459(0.261,0.806) | 0.012 |
| Q4(1.4-1.62) | 132/729 | 0.489(0.373,0.641) | | <0.001 |  | 0.360(0.237,0.547) | <0.001 |  | 0.377(0.247,0.575) | <0.001 |
| Q5(>1.62) | 140/732 | 0.726(0.509,1.034) | | 0.074 |  | 0.558(0.322,0.968) | 0.040 |  | 0.606(0.348,1.056) | 0.072 |
| **Per 1SD change** |  | 0.913(0.819,1.016) | | 0.093 |  | 0.822(0.692,0.978) | 0.029 |  | 0.850(0.713,1.013) | 0.067 |
| ***p* for trend** |  |  | | 0.042 |  |  | 0.014 |  |  | 0.030 |
| **AA(n6),%** |  |  | |  |  |  |  |  |  |  |
| Q1(<5.74) | 182/735 | 1(Ref) | |  |  | 1(Ref) |  |  | 1(Ref) |  |
| Q2(5.74-6.77) | 133/763 | 0.526(0.361,0.766) | | 0.002 |  | 0.552(0.382,0.798) | 0.004 |  | 0.583(0.400,0.849) | 0.010 |
| Q3(6.77-7.72) | 139/755 | 0.562(0.371,0.852) | | 0.008 |  | 0.582(0.354,0.957) | 0.035 |  | 0.593(0.350,1.005) | 0.052 |
| Q4(7.72-8.91) | 142/760 | 0.489(0.355,0.675) | | <0.001 |  | 0.435(0.293,0.647) | 0.001 |  | 0.433(0.282,0.666) | 0.001 |
| Q5(>8.91) | 173/746 | 0.596(0.376,0.945) | | 0.029 |  | 0.432(0.246,0.757) | 0.007 |  | 0.396(0.232,0.674) | 0.003 |
| **Per 1SD change** |  | 0.823(0.695,0.974) | | 0.025 |  | 0.721(0.587,0.886) | 0.004 |  | 0.696(0.576,0.842) | 0.001 |
| ***p* for trend** |  |  | | 0.024 |  |  | 0.004 |  |  | 0.002 |
| **EDA(n6),%** |  |  | |  |  |  |  |  |  |  |
| Q1(<0.16) | 140/691 |  | |  |  |  |  |  |  |  |
| Q2(0.16-0.18) | 133/713 | 0.953(0.696,1.305) | | 0.758 |  | 0.917(0.583,1.442) | 0.685 |  | 0.956(0.608,1.502) | 0.829 |
| Q3(0.18-0.2) | 139/705 | 0.862(0.655,1.136) | | 0.280 |  | 0.752(0.493,1.149) | 0.170 |  | 0.766(0.487,1.205) | 0.219 |
| Q4(0.2-0.23) | 170/690 | 1.242(0.959,1.609) | | 0.098 |  | 1.125(0.751,1.687) | 0.540 |  | 1.187(0.810,1.740) | 0.342 |
| Q5(>0.23) | 132/734 | 0.905(0.654,1.252) | | 0.534 |  | 0.811(0.447,1.472) | 0.460 |  | 0.839(0.454,1.549) | 0.537 |
| **Per 1SD change** |  | 1.024(0.924,1.135) | | 0.638 |  | 0.980(0.806,1.193) | 0.833 |  | 0.994(0.816,1.210) | 0.945 |
| ***p* for trend** |  |  | | 0.823 |  |  | 0.734 |  |  | 0.850 |
| **DTA(n6),%** |  |  | |  |  |  |  |  |  |  |
| Q1(<0.18) | 152/728 | 1(Ref) | |  |  | 1(Ref) |  |  | 1(Ref) |  |
| Q2(0.18-0.21) | 154/738 | 1.123(0.809,1.558) | | 0.474 |  | 0.748(0.503,1.111) | 0.137 |  | 0.728(0.479,1.104) | 0.120 |
| Q3(0.21-0.24) | 147/748 | 1.456(1.071,1.980) | | 0.018 |  | 0.914(0.625,1.336) | 0.617 |  | 0.899(0.617,1.310) | 0.544 |
| Q4(0.24-0.28) | 137/727 | 1.248(0.835,1.865) | | 0.268 |  | 0.690(0.379,1.254) | 0.202 |  | 0.639(0.344,1.187) | 0.138 |
| Q5(>0.28) | 166/751 | 1.360(0.819,2.258) | | 0.225 |  | 0.617(0.307,1.238) | 0.158 |  | 0.545(0.257,1.155) | 0.102 |
| **Per 1SD change** |  | 1.128(0.974,1.305) | | 0.104 |  | 0.868(0.679,1.110) | 0.240 |  | 0.835(0.648,1.076) | 0.148 |
| ***p* for trend** |  |  | | 0.197 |  |  | 0.213 |  |  | 0.132 |
| **DPAn6(n6),%** |  |  | |  |  |  |  |  |  |  |
| Q1(<0.13) | 264/725 | 1(Ref) | |  |  | 1(Ref) |  |  | 1(Ref) |  |
| Q2(0.13-0.16) | 198/740 | 1.086(0.705,1.671) | | 0.699 |  | 0.776(0.421,1.430) | 0.387 |  | 0.762(0.406,1.432) | 0.360 |
| Q3(0.16-0.19) | 131/750 | 0.865(0.537,1.395) | | 0.539 |  | 0.686(0.355,1.328) | 0.240 |  | 0.667(0.337,1.321) | 0.216 |
| Q4(0.19-0.23) | 115/755 | 1.045(0.613,1.781) | | 0.868 |  | 0.781(0.354,1.722) | 0.511 |  | 0.755(0.321,1.772) | 0.479 |
| Q5(>0.23) | 61/785 | 1.308(0.850,2.013) | | 0.213 |  | 0.802(0.413,1.557) | 0.485 |  | 0.736(0.383,1.414) | 0.320 |
| **Per 1SD change** |  | 1.057(0.901,1.240) | | 0.485 |  | 0.889(0.699,1.131) | 0.316 |  | 0.859(0.683,1.080) | 0.176 |
| ***p* for trend** |  |  | | 0.399 |  |  | 0.518 |  |  | 0.381 |

Model1: adjusted for age;

Model2: adjusted for age, sex, race, education level, BMI, waist circumference, family income-to-poverty ratio, smoking status, drinking status and physical activity;

Model3: adjusted for age, sex, race, education level, BMI, waist circumference, family income-to-poverty ratio, smoking status, drinking status and physical activity, hypertension, cardiovascular disease and cancer.

Table S20. Adjusted OR for associations of different n-5 and n-7 PUFAs with the prevalence of T2DM

|  | Diabetes | | | | | | | | | |
| --- | --- | --- | --- | --- | --- | --- | --- | --- | --- | --- |
|  |  | | Model1 | |  | Model2 | |  | Model3 | |
|  | Case/N | OR(95% CI) | | *P*-value |  | OR(95% CI) | *P*-value |  | OR(95% CI) | *P*-value |
| **MA(n5),%** |  |  | |  |  |  |  |  |  |  |
| Q1(<0.03) | 139/726 | 1(Ref) | |  |  | 1(Ref) |  |  | 1(Ref) |  |
| Q2(0.03-0.04) | 130/701 | 0.841(0.593,1.194) | | 0.319 |  | 0.768(0.476,1.237) | 0.252 |  | 0.840(0.493,1.432) | 0.484 |
| Q3(0.04-0.06) | 149/715 | 1.060(0.648,1.736) | | 0.809 |  | 1.141(0.656,1.986) | 0.615 |  | 1.224(0.689,2.177) | 0.451 |
| Q4(0.06-0.09) | 159/692 | 1.527(0.928,2.512) | | 0.093 |  | 1.519(0.882,2.615) | 0.121 |  | 1.658(0.915,3.005) | 0.087 |
| Q5(>0.09) | 137/699 | 1.274(0.782,2.076) | | 0.318 |  | 1.376(0.789,2.400) | 0.237 |  | 1.445(0.803,2.600) | 0.193 |
| **Per 1SD change** |  | 1.081(0.949,1.230) | | 0.232 |  | 1.124(0.957,1.319) | 0.143 |  | 1.131(0.963,1.328) | 0.121 |
| ***p* for trend** |  |  | | 0.064 |  |  | 0.033 |  |  | 0.032 |
| **PA(n7),%** |  |  | |  |  |  |  |  |  |  |
| Q1(<1.24) | 135/769 | 1(Ref) | |  |  | 1(Ref) |  |  | 1(Ref) |  |
| Q2(1.24-1.61) | 125/769 | 0.999(0.731,1.366) | | 0.996 |  | 0.966(0.574,1.628) | 0.889 |  | 0.966(0.539,1.732) | 0.898 |
| Q3(1.61-2.02) | 133/754 | 1.060(0.726,1.548) | | 0.756 |  | 0.951(0.495,1.826) | 0.870 |  | 0.906(0.459,1.789) | 0.754 |
| Q4(2.02-2.58) | 177/719 | 1.551(1.009,2.384) | | 0.046 |  | 1.602(0.826,3.109) | 0.148 |  | 1.627(0.801,3.305) | 0.157 |
| Q5(>2.58) | 197/720 | 1.922(1.272,2.904) | | 0.003 |  | 1.553(0.988,2.440) | 0.055 |  | 1.466(0.886,2.425) | 0.121 |
| **Per 1SD change** |  | 1.202(1.085,1.333) | | 0.001 |  | 1.169(1.041,1.312) | 0.012 |  | 1.154(1.024,1.300) | 0.023 |
| ***p* for trend** |  |  | | 0.001 |  |  | 0.006 |  |  | 0.015 |
| **VA(n7),%** |  |  | |  |  |  |  |  |  |  |
| Q1(<1.08) | 121/725 | 1(Ref) | |  |  | 1(Ref) |  |  | 1(Ref) |  |
| Q2(1.08-1.21) | 144/736 | 1.003(0.641,1.570) | | 0.988 |  | 0.808(0.519,1.256) | 0.316 |  | 0.793(0.521,1.208) | 0.248 |
| Q3(1.21-1.34) | 123/727 | 0.71(0.468,1.079) | | 0.105 |  | 0.619(0.355,1.077) | 0.084 |  | 0.606(0.343,1.068) | 0.077 |
| Q4(1.34-1.5) | 169/747 | 0.991(0.660,1.489) | | 0.964 |  | 0.841(0.543,1.302) | 0.407 |  | 0.808(0.500,1.307) | 0.347 |
| Q5(>1.5) | 200/742 | 1.137(0.802,1.614) | | 0.457 |  | 0.904(0.570,1.434) | 0.644 |  | 0.79(0.477,1.307) | 0.321 |
| **Per 1SD change** |  | 1.054(0.966,1.150) | | 0.226 |  | 0.988(0.875,1.117) | 0.843 |  | 0.957(0.845,1.083) | 0.453 |
| ***p* for trend** |  |  | | 0.470 |  |  | 0.821 |  |  | 0.414 |

Model1: adjusted for age;

Model2: adjusted for age, sex, race, education level, BMI, waist circumference, family income-to-poverty ratio, smoking status, drinking status and physical activity;

Model3: adjusted for age, sex, race, education level, BMI, waist circumference, family income-to-poverty ratio, smoking status, drinking status and physical activity, hypertension, cardiovascular disease and cancer.

Table S21. Adjusted OR for associations of different n-9 PUFAs with the prevalence of T2DM

|  | Diabetes | | | | | | | | |
| --- | --- | --- | --- | --- | --- | --- | --- | --- | --- |
|  |  | | Model1 |  | Model2 | |  | Model3 | |
|  | Case/N | OR(95% CI) | *P*-value |  | OR(95% CI) | *P*-value |  | OR(95% CI) | *P*-value |
| **OA(n9),%** |  |  |  |  |  |  |  |  |  |
| Q1(<15.96) | 85/767 | 1(Ref) |  |  | 1(Ref) |  |  | 1(Ref) |  |
| Q2(15.96-17.51) | 114/746 | 1.505(0.98,2.313) | 0.061 |  | 1.956(1.112,3.441) | 0.023 |  | 1.866(1.043,3.340) | 0.038 |
| Q3(17.51-19.13) | 142/756 | 1.530(0.911,2.571) | 0.104 |  | 1.644(0.826,3.273) | 0.142 |  | 1.722(0.868,3.415) | 0.108 |
| Q4(19.13-20.97) | 173/727 | 1.872(1.316,2.664) | 0.001 |  | 2.081(1.342,3.226) | 0.003 |  | 2.095(1.387,3.165) | 0.003 |
| Q5(>20.97) | 254/753 | 3.599(2.363,5.481) | <0.001 |  | 3.776(2.314,6.162) | <0.001 |  | 3.541(2.162,5.799) | <0.001 |
| **Per 1SD change** |  | 1.593(1.404,1.807) | <0.001 |  | 1.591(1.398,1.811) | <0.001 |  | 1.552(1.360,1.772) | <0.001 |
| ***p* for trend** |  |  | <0.001 |  |  | <0.001 |  |  | <0.001 |
| **EA(n9),%** |  |  |  |  |  |  |  |  |  |
| Q1(<0.1) | 101/687 | 1(Ref) |  |  | 1(Ref) |  |  | 1(Ref) |  |
| Q2(0.1-0.11) | 116/713 | 1.085(0.738,1.595) | 0.668 |  | 1.267(0.782,2.053) | 0.308 |  | 1.215(0.733,2.013) | 0.410 |
| Q3(0.11-0.13) | 149/710 | 1.187(0.786,1.791) | 0.401 |  | 1.470(0.807,2.681) | 0.189 |  | 1.454(0.815,2.596) | 0.181 |
| Q4(0.13-0.15) | 158/719 | 1.576(1.090,2.281) | 0.018 |  | 1.993(1.121,3.542) | 0.022 |  | 1.814(1.014,3.246) | 0.046 |
| Q5(>0.15) | 194/740 | 1.913(1.282,2.855) | 0.003 |  | 2.177(1.202,3.945) | 0.014 |  | 2.070(1.097,3.904) | 0.029 |
| **Per 1SD change** |  | 1.291(1.128,1.478) | 0.001 |  | 1.302(1.042,1.626) | 0.023 |  | 1.287(1.022,1.621) | 0.034 |
| ***p* for trend** |  |  | <0.001 |  |  | 0.003 |  |  | 0.008 |
| **NRA(n9),%** |  |  |  |  |  |  |  |  |  |
| Q1(<0.56) | 213/682 | 1(Ref) |  |  | 1(Ref) |  |  | 1(Ref) |  |
| Q2(0.56-0.68) | 170/698 | 0.690(0.421,1.130) | 0.134 |  | 0.843(0.512,1.387) | 0.472 |  | 0.839(0.515,1.367) | 0.441 |
| Q3(0.68-0.79) | 116/697 | 0.355(0.218,0.577) | <0.001 |  | 0.426(0.270,0.672) | 0.001 |  | 0.432(0.263,0.711) | 0.004 |
| Q4(0.79-0.92) | 104/700 | 0.335(0.238,0.471) | <0.001 |  | 0.360(0.227,0.572) | <0.001 |  | 0.368(0.227,0.597) | 0.001 |
| Q5(>0.92) | 102/699 | 0.277(0.158,0.487) | <0.001 |  | 0.316(0.175,0.570) | 0.001 |  | 0.318(0.171,0.593) | 0.002 |
| **Per 1SD change** |  | 0.554(0.462,0.663) | <0.001 |  | 0.579(0.483,0.695) | <0.001 |  | 0.581(0.481,0.702) | <0.001 |
| ***p* for trend** |  |  | <0.001 |  |  | <0.001 |  |  | <0.001 |

Model1: adjusted for age;

Model2: adjusted for age, sex, race, education level, BMI, waist circumference, family income-to-poverty ratio, smoking status, drinking status and physical activity;

Model3: adjusted for age, sex, race, education level, BMI, waist circumference, family income-to-poverty ratio, smoking status, drinking status and physical activity, hypertension, cardiovascular disease and cancer.

Table S22. Adjusted OR for associations of total n3, n6 n9 with the prevalence of T2DM

|  |  | T2DM | |
| --- | --- | --- | --- |
|  | Case/N | OR(95% CI) ^*^ | *P*-value |
| **Total n3,%** |  |  |  |
| Q1(<2.20) | 139/765 | 1(Ref) |  |
| Q2(2.20-2.54) | 121/757 | 0.899(0.48,1.681) | 0.711 |
| Q3(2.54-2.91) | 165/769 | 1.016(0.697,1.483) | 0.926 |
| Q4(2.91-3.53) | 158/740 | 0.884(0.498,1.571) | 0.644 |
| Q5(>3.53) | 187/729 | 1.072(0.731,1.571) | 0.693 |
| **Per 1SD change** |  | 0.958(0.815,1.125) | 0.573 |
| ***p* for trend** |  |  | 0.831 |
| **Total n6,%** |  |  |  |
| Q1(<37.19) | 260/736 | 1(Ref) |  |
| Q2(37.19-40.35) | 205/732 | 0.733(0.483,1.114) | 0.13 |
| Q3(40.35-42.89) | 126/737 | 0.45(0.271,0.749) | 0.006 |
| Q4(42.89-45.54) | 109/776 | 0.324(0.191,0.547) | 0.001 |
| Q5(>45.54) | 70/779 | 0.174(0.091,0.33) | <0.001 |
| **Per 1SD change** |  | 0.591(0.504,0.693) | <0.001 |
| ***p* for trend** |  |  | <0.001 |
| **Total n9,%** |  |  |  |
| Q1(<16.86) | 86/773 | 1(Ref) |  |
| Q2(16.86-18.43) | 105/743 | 1.845(1.101,3.093) | 0.025 |
| Q3(18.43-19.99) | 152/755 | 1.74(0.946,3.2) | 0.07 |
| Q4(19.99-21.73) | 171/733 | 1.952(1.203,3.169) | 0.012 |
| Q5(>21.73) | 256/756 | 3.526(2.161,5.753) | <0.001 |
| **Per 1SD change** |  | 1.482(1.267,1.734) | <0.001 |
| ***p* for trend** |  |  | <0.001 |

*adjusted for age, sex, race, education level, BMI, waist circumference, family income-to-poverty ratio, smoking status, drinking status and physical activity, hypertension, cardiovascular disease and cancer.

**SUPPLEMENTAL FIGURES**

**Figure S1.**

**
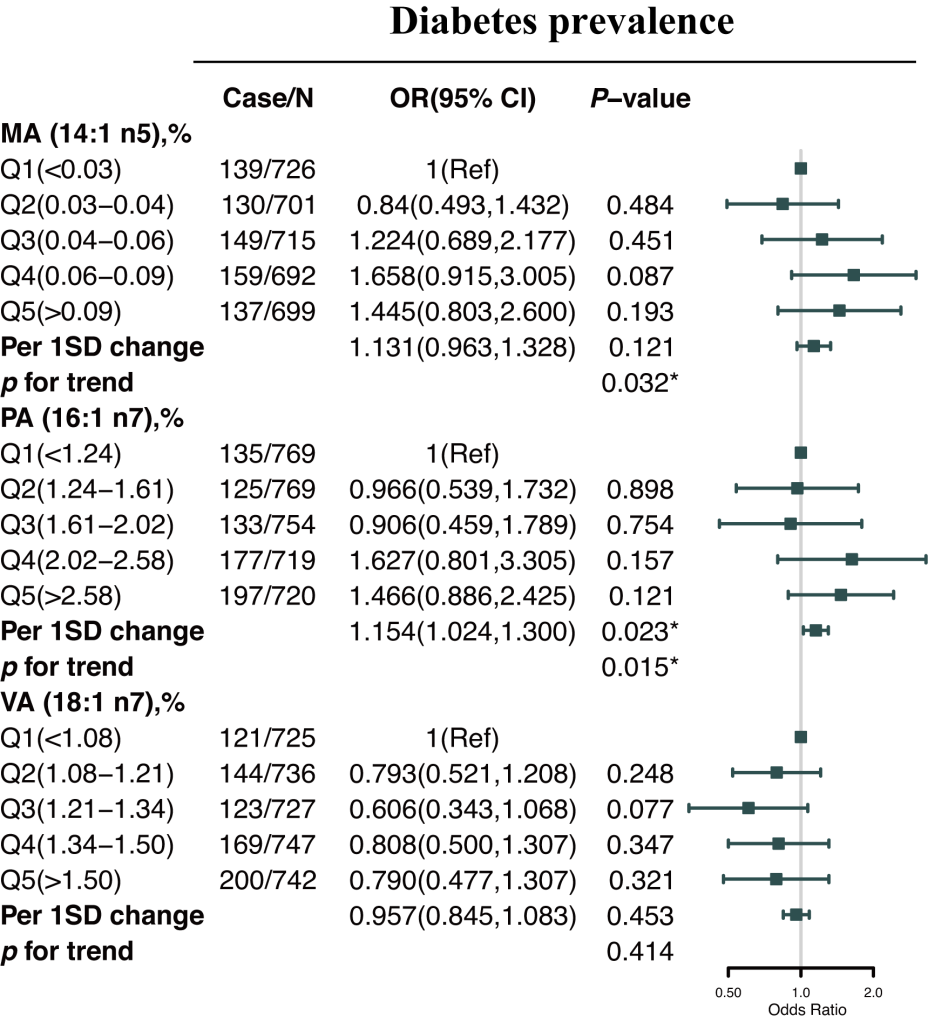
**

**Figure S2.**

**
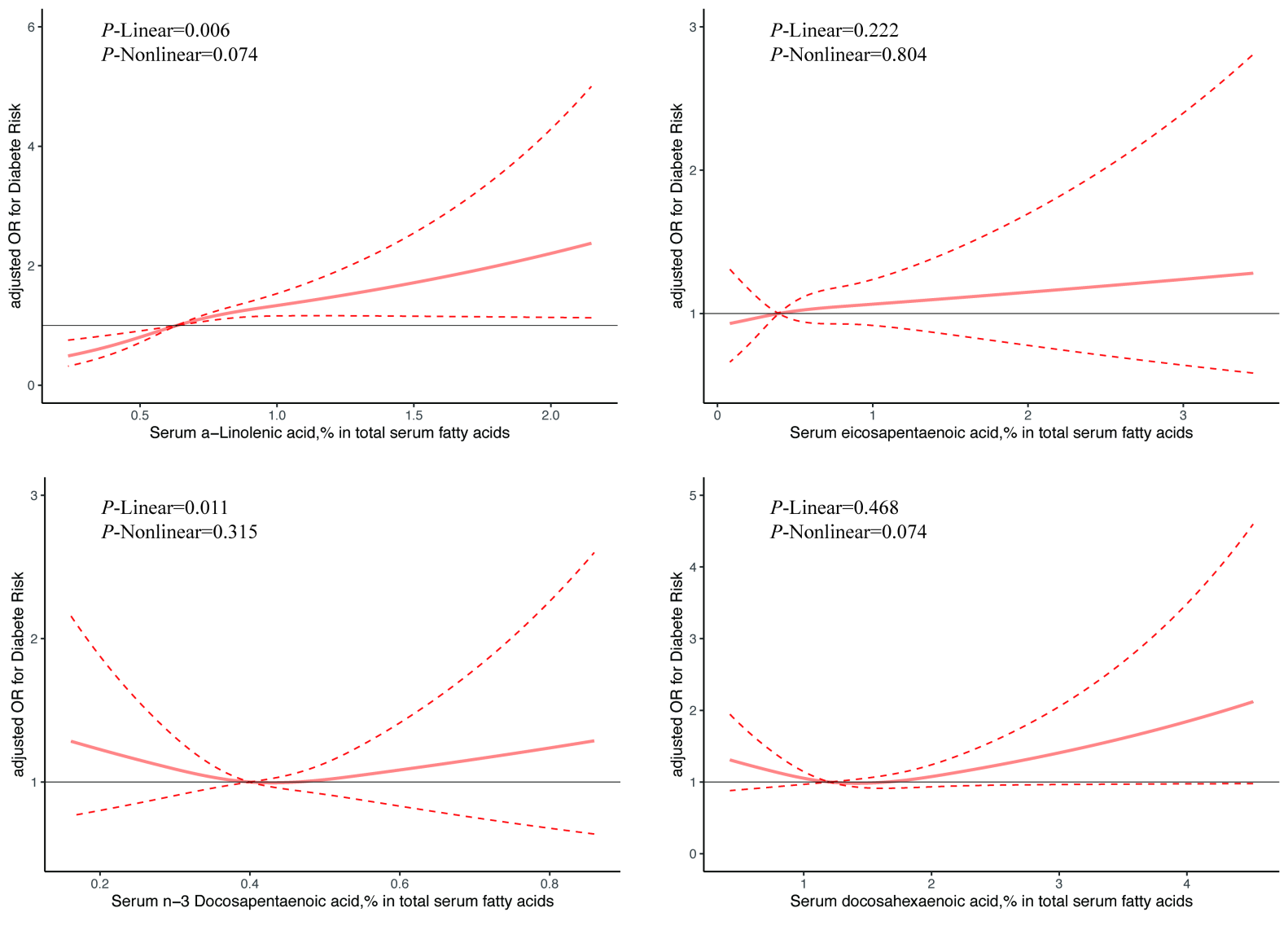
**

**Figure S3.**


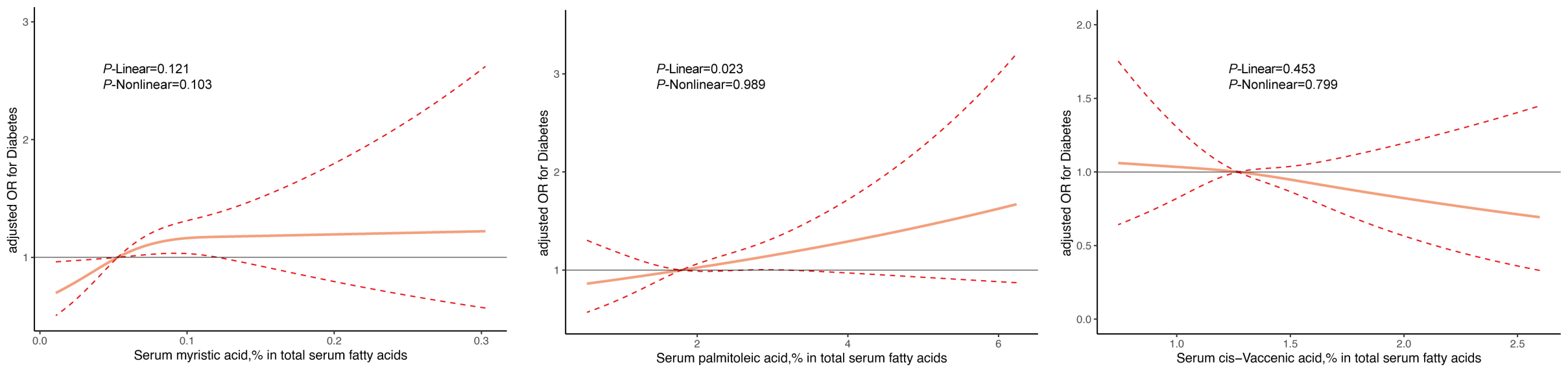


**Figure S4.**


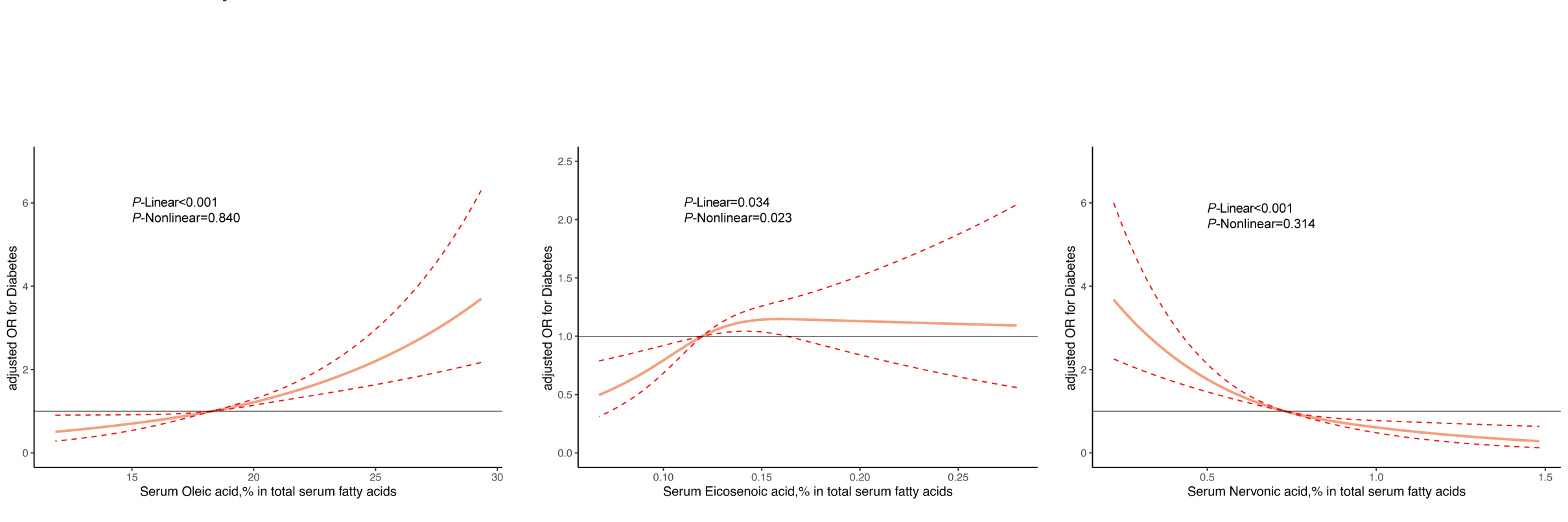

Supplement: Supplementary file 1 — Supporting information Additional supporting information can be found online in the Supporting Information section. Includes the baseline characteristics according to quintiles of serum PUFAs and MUFAs (Table S1–Table S17), adjusted OR for associations of PUFAs and MUFAs with the prevalence of T2DM (Table S18–Table S22). Adjusted OR for associations of different n‐5 and n‐7 PUFAs with the prevalence of T2DM (Figure S1), associations of n‐3 PUFAs with the prevalence of T2DM (Figure S2), associations of serum MA (14:1 n5), PA (16:1 n7), and VA (18:1 n7) with the prevalence of T2DM (Figure S3), associations of n‐9 MUFAs with the prevalence of T2DM (Figure S4). [file JDR-2026-1153035-s001.docx]
